# Mitigation of Environmental Stress-Impacts in Plants: Role of Sole and Combinatory Exogenous Application of Glutathione

**DOI:** 10.3389/fpls.2021.791205

**Published:** 2021-12-22

**Authors:** Yi Sze Koh, See Kiat Wong, Nor Hadiani Ismail, Gokhan Zengin, Acharaporn Duangjai, Surasak Saokaew, Pochamana Phisalprapa, Khang Wei Tan, Bey Hing Goh, Siah Ying Tang

**Affiliations:** ^1^Chemical Engineering Discipline, School of Engineering, Monash University Malaysia, Subang Jaya, Malaysia; ^2^Faculty of Applied Sciences, Universiti Teknologi MARA (UiTM), Shah Alam, Malaysia; ^3^Atta-ur-Rahman Institute for Natural Product Discovery (AuRIns), Universiti Teknologi MARA (UiTM), Puncak Alam, Malaysia; ^4^Department of Biology, Science Faculty, Selcuk University, Konya, Turkey; ^5^Unit of Excellence in Research and Product Development of Coffee, Division of Physiology, School of Medical Sciences, University of Phayao, Mae Ka, Thailand; ^6^Center of Health Outcomes Research and Therapeutic Safety (Cohorts), School of Pharmaceutical Sciences, University of Phayao, Mae Ka, Thailand; ^7^Unit of Excellence on Clinical Outcomes Research and IntegratioN (UNICORN), School of Pharmaceutical Sciences, University of Phayao, Mae Ka, Thailand; ^8^Unit of Excellence on Herbal Medicine, School of Pharmaceutical Sciences, University of Phayao, Mae Ka, Thailand; ^9^Department of Pharmaceutical Care, Division of Pharmacy Practice, School of Pharmaceutical Sciences, University of Phayao, Mae Ka, Thailand; ^10^Department of Medicine, Division of Ambulatory Medicine, Faculty of Medicine Siriraj Hospital, Mahidol University, Salaya, Thailand; ^11^School of Energy and Chemical Engineering, Xiamen University Malaysia, Sepang, Malaysia; ^12^Biofunctional Molecule Exploratory Research Group, School of Pharmacy, Monash University Malaysia, Subang Jaya, Malaysia; ^13^College of Pharmaceutical Sciences, Zhejiang University, Hangzhou, China; ^14^Tropical Medicine and Biology Platform, School of Science, Monash University Malaysia, Subang Jaya, Malaysia

**Keywords:** biostimulant, cyanobacteria, environmental stresses, glutathione, plant metabolite

## Abstract

Glutathione (GSH; γ-glutamyl-cysteinyl-glycine), a low-molecular-weight thiol, is the most pivotal metabolite involved in the antioxidative defense system of plants. The modulation of GSH on the plant in response to environmental stresses could be illustrated through key pathways such as reactive oxygen species (ROS) scavenging and signaling, methylglyoxal (MG) detoxification and signaling, upregulation of gene expression for antioxidant enzymes, and metal chelation and xenobiotic detoxification. However, under extreme stresses, the biosynthesis of GSH may get inhibited, causing an excess accumulation of ROS that induces oxidative damage on plants. Hence, this gives rise to the idea of exploring the use of exogenous GSH in mitigating various abiotic stresses. Extensive studies conducted borne positive results in plant growth with the integration of exogenous GSH. The same is being observed in terms of crop yield index and correlated intrinsic properties. Though, the improvement in plant growth and yield contributed by exogenous GSH is limited and subjected to the glutathione pool [GSH/GSSG; the ratio of reduced glutathione (GSH) to oxidized glutathione (GSSG)] homeostasis. Therefore, recent studies focused on the sequenced application of GSH was performed in order to complement the existing limitation. Along with various innovative approaches in combinatory use with different bioactive compounds (proline, citric acid, ascorbic acid, melatonin), biostimulants (putrescine, *Moringa* leaf extract, selenium, humic acid), and microorganisms (cyanobacteria) have resulted in significant improvements when compared to the individual application of GSH. In this review, we reinforced our understanding of biosynthesis, metabolism and consolidated different roles of exogenous GSH in response to environmental stresses. Strategy was also taken by focusing on the recent progress of research in this niche area by covering on its individualized and combinatory applications of GSH prominently in response to the abiotic stresses. In short, the review provides a holistic overview of GSH and may shed light on future studies and its uses.

## Introduction

Environmental stresses caused by the worsening global warming and climate changes remain one of the biggest challenges in the agricultural sector, significantly limiting the productivity of crops (Jalil and Ansari, 2020; Chaudhry and Sidhu, 2021). As a result of the environmental changes, abiotic stresses such as temperature extremities, drought, salinity, and metal toxicity, have contributed to the reduction of yield as high as 50% for major crops (Stavridou et al., 2018; Francini and Sebastiani, 2019). Due to their sessile nature, plants have various innate mechanisms that generate specific cellular responses to mitigate abiotic stresses (Yadav et al., 2020). Toward improving crop yield and productivity, changes in physiological, biochemical, and metabolic processes of plants affected by the abiotic stresses and their response mechanisms have been studied extensively (Hasanuzzaman et al., 2017; Wilma Sabetta et al., 2017; Debnath et al., 2021).

Generally, abiotic stresses cause excessive production of reactive oxygen species (ROS), either in molecular states like hydrogen peroxide (H_2_O_2_) and singlet oxygen (^1^O_2_) or in ionic states such as hydroxyl radicals (OḤ) and superoxide anions ($O_{2}^{\cdot -}$; Huang et al., 2019). Under normal conditions, ROS is generated as a by-product of aerobic metabolism at basal levels, confined to cellular compartments like mitochondria, chloroplasts, peroxisomes, and scavenged by antioxidant mechanisms, to prevent any damage to the plants. However, under stressed conditions, the overproduction of ROS causes oxidative damage to proteins, lipids, and deoxyribonucleic acid (DNA), possibly leading to plant death (Das and Roychoudhury, 2014). In response, plants rely on their antioxidative defense system, which consists of the enzymatic and non-enzymatic antioxidants, to regulate the ROS, improving the stress tolerance in plants (Hasanuzzaman et al., 2017).

Among the numerous antioxidants, glutathione (GSH; γ-glutamyl-cysteinyl-glycine) has been known to be one of the most abundant and essential metabolites in the defense system to combat abiotic stresses (Locato et al., 2017). Glutathione is usually present in its reduced form, GSH, and acts as a ROS scavenger, where it is oxidized into glutathione disulfide (GSSG; Wilma Sabetta et al., 2017). Under the normal level of stresses, biosynthesis of GSH is induced to maintain the GSH/GSSG ratio (Locato et al., 2017). However, under extreme stresses, the biosynthesis of GSH may be inhibited, causing an imbalance and a significant decrease in the GSH/GSSG ratio. Thus, leading to the inability to scavenge the overproduced ROS, which will inflict oxidative damage to the plants (Cao et al., 2017). Therefore, studies have been focused on investigating the effects of exogenous application of GSH on plants *via* foliar spray or seed soaking method to compensate for the decreased level of endogenous GSH, which in an attempt to alter the redox state of GSH/GSSG. Positive results for the exogenous application of GSH were shown especially in mitigating abiotic stresses like drought, salinity, metal toxicity, and temperature extremities and further improving the growth and yield of major plant crops that include rice, wheat, maize, and chickpea (Hussain et al., 2016; El-Beltagi et al., 2020; Li et al., 2021; Wang et al., 2021).

However, the improvement of plant growth parameters contributed by the exogenous application of GSH is limited and subject to the ratio of the oxidized and reduced glutathione, GSH/GSSG. Studies show that a higher dosage of GSH did not correlate to the best growth parameters for all plant species. For example, among concentrations (5 mM, 10 mM, 15 mM and 20 mM), 10 mM of GSH resulted in the best growth parameters for rice species MR 253, while 20 mM of GSH resulted in the best growth parameters for rice species MR220 (Teh et al., 2015). Hence, in recent years, researchers have started to investigate the sequenced or combined application of GSH together with different bioactive compounds, categorized as plant metabolites such as proline, citric acid, ascorbic acid, melatonin; biostimulants such as putrescine, *Moringa* leaf extract, selenium and humic acid; and microorganism like cyanobacteria, in attempt to improve the stress tolerance further and overcome the limitations of possessed by GSH in conferring growth and yield of plants (Semida et al., 2018; Zaki et al., 2019; Goodarzi et al., 2020; Rehman et al., 2021; Zeng et al., 2021). Therefore, this review discusses the recent progress (2017–2021) in the individual and combined application of GSH with other bioactive compounds while reinforcing our understanding of the biosynthesis, metabolism, and roles of GSH when mitigating abiotic and biotic stresses.

## Glutathione

### Origin

De Rey-Pailhade first discovered glutathione in 1888 from extracts of yeast, egg whites and many other animal tissues (Alanazi et al., 2015). Due to the discovery of glutathione’s functions in human body fluids, studies on its structure and functions began extensively in the 1960s. At the same time, its metabolism was confirmed in 1983 by Dr. Alton Meister (Meister, 1988). Roles of glutathione (GSH) as a plant growth regulator along with its structures and metabolism are elaborated in the sections below.

### Localization

GSH, a low-molecular-weight thiol, is found innately and abundantly in plant tissues, typically present in millimolar concentrations (Wilma Sabetta et al., 2017). Generally, GSH is found in the roots and leaves of plants, with the highest concentrations primarily found in the mitochondria, followed by nuclei, peroxisomes, cytosol, plastids, and trace concentrations found in chloroplast and vacuoles (Hasanuzzaman et al., 2012; Wilma Sabetta et al., 2017).

### Biochemical and Molecular Characterization

**Figure 1:**
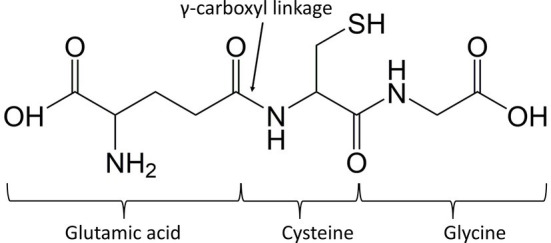
Chemical structure of glutathione (GSH), a tripeptide made up of glutamic acid, cysteine, and glycine.

Chemically speaking, GSH is a tripeptide composed of three amino acids which are glutamate (or glutamic acid), cysteine and glycine, making up γ-glutamylcysteinylglycine (γ-Glu-Cys-Gly) as shown in Figure 1 (Wilma Sabetta et al., 2017). GSH has a unique bond between glutamate and cysteine. The link occurs through the γ-carboxyl group instead of the expected α-carboxyl group of glutamate, making the molecule stable, which will not be degraded by aminopeptidases (Lu, 2013). It is soluble in water due to the presence of hydrophilic groups, consisting of two carboxylic groups (COOH), one amine group (NH) and one thiol group (SH). Overall, the most chemically reactive group of GSH is the thiol (SH) group in cysteine. It is responsible for most biochemical functions like redox reactions and nucleophilic displacements, which are essential to carry out the detoxification of xenobiotics (Hasanuzzaman et al., 2017).

### Biosynthesis and Redox Regulation

The biosynthesis of GSH occurs in a two-step reaction involving two adenosine triphosphate (ATP) molecules catalyzed by two enzymes, namely, γ-glutamylcysteine synthetase (γ-ECS) and glutathione synthetase (GSHS). First, the γ-ECS catalyzes the formation of an intermediate product γ-glutamylcysteine (γ-EC) and acts as a ligase between the amino acid glutamate and cysteine. In the second step, GSHS catalyzes the synthesis of another intermediate product, acylphosphate, at the C-terminal of the first intermediate γ-EC. The biosynthesis is followed by displacing an inorganic phosphate linking the third amino acid glycine, producing GSH (Musgrave et al., 2013; Wilma Sabetta et al., 2017). γ-ECS have been found primarily on chloroplasts, a type of plastid. In contrast, GSHS has been found in both cytosol and plastids, proving that the biosynthesis of GSH is a compartmentalized process. γ-EC may also move from the chloroplast or plastid to the cytosol as a substrate of GSHS to carry out the biosynthesis of GSH in the cytosol. Upon completion, GSH can either be transported back into the chloroplast or to the mitochondria (Hasanuzzaman et al., 2017).

**Figure 2:**
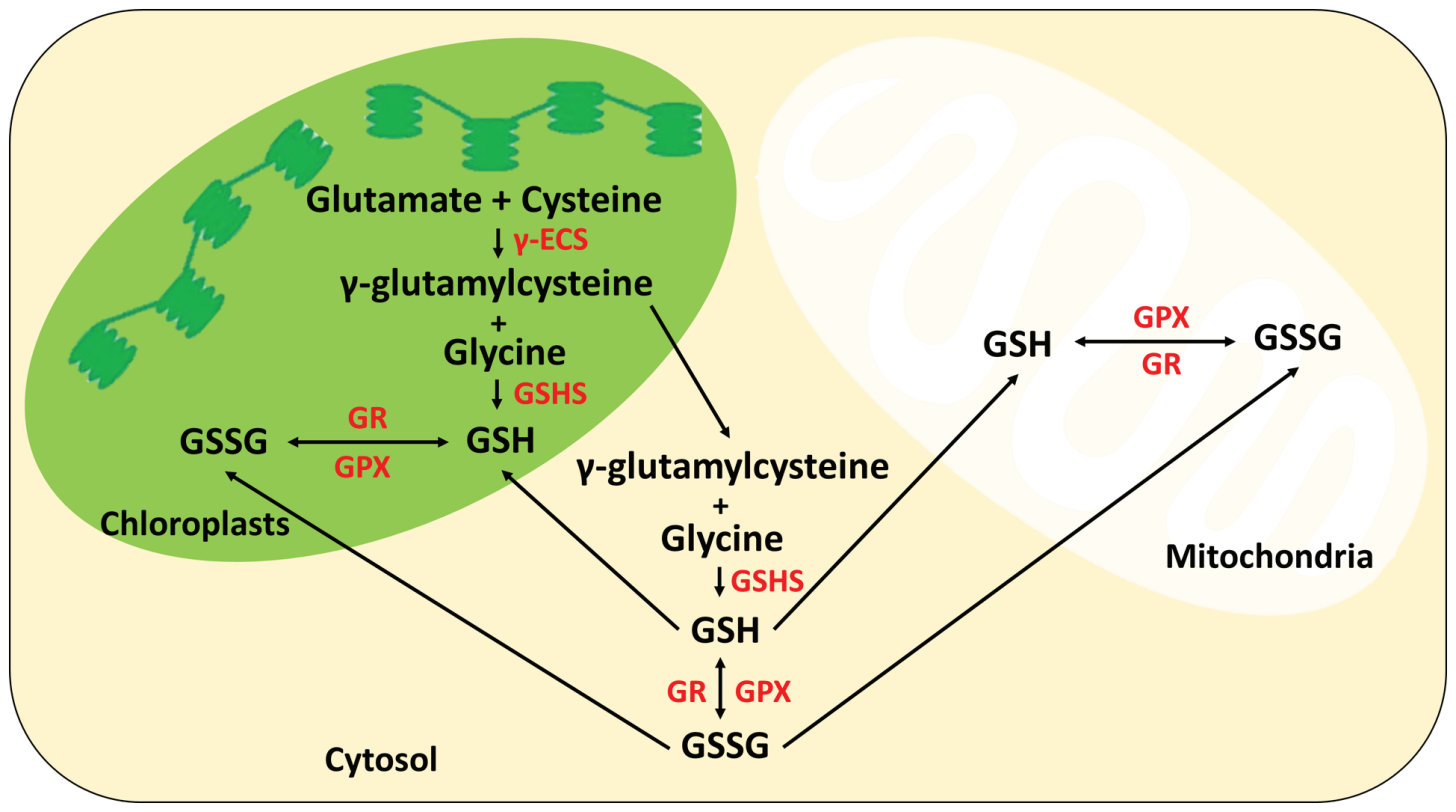
Illustration of biosynthesis and simple metabolism of GSH in plant organelles.

The glutathione system, comprising the reduced form, GSH and oxidized form, GSSG, plays a crucial role in maintaining the redox balance in plants (Bela et al., 2017a). During stressed conditions, the enzyme glutathione peroxidase (GPX) uses GSH as a reducing agent to remove excess H_2_O_2_ induced by abiotic stresses, into the oxidized glutathione, GSSG. In defense against the overproduced ROS during stresses, the enzyme glutathione reductase (GR) then reduces GSSG back into GSH, further maintaining the GSH/GSSG homeostasis (Cao et al., 2017; Bela et al., 2017b). Evidently, various studies showed up-regulation of genes encoding GPX and a significant increase in GR activities in plants that were subjected to abiotic stresses such as metal toxicity, salinity, drought, and temperature extremities (Nahar et al., 2015; Ding et al., 2016; Hussain et al., 2016; Li et al., 2021). The overall biosynthesis of GSH in the chloroplasts, mitochondria, and cytosol is shown in Figure 2.

### Modulation in Stressed Plants

**Figure 3:**
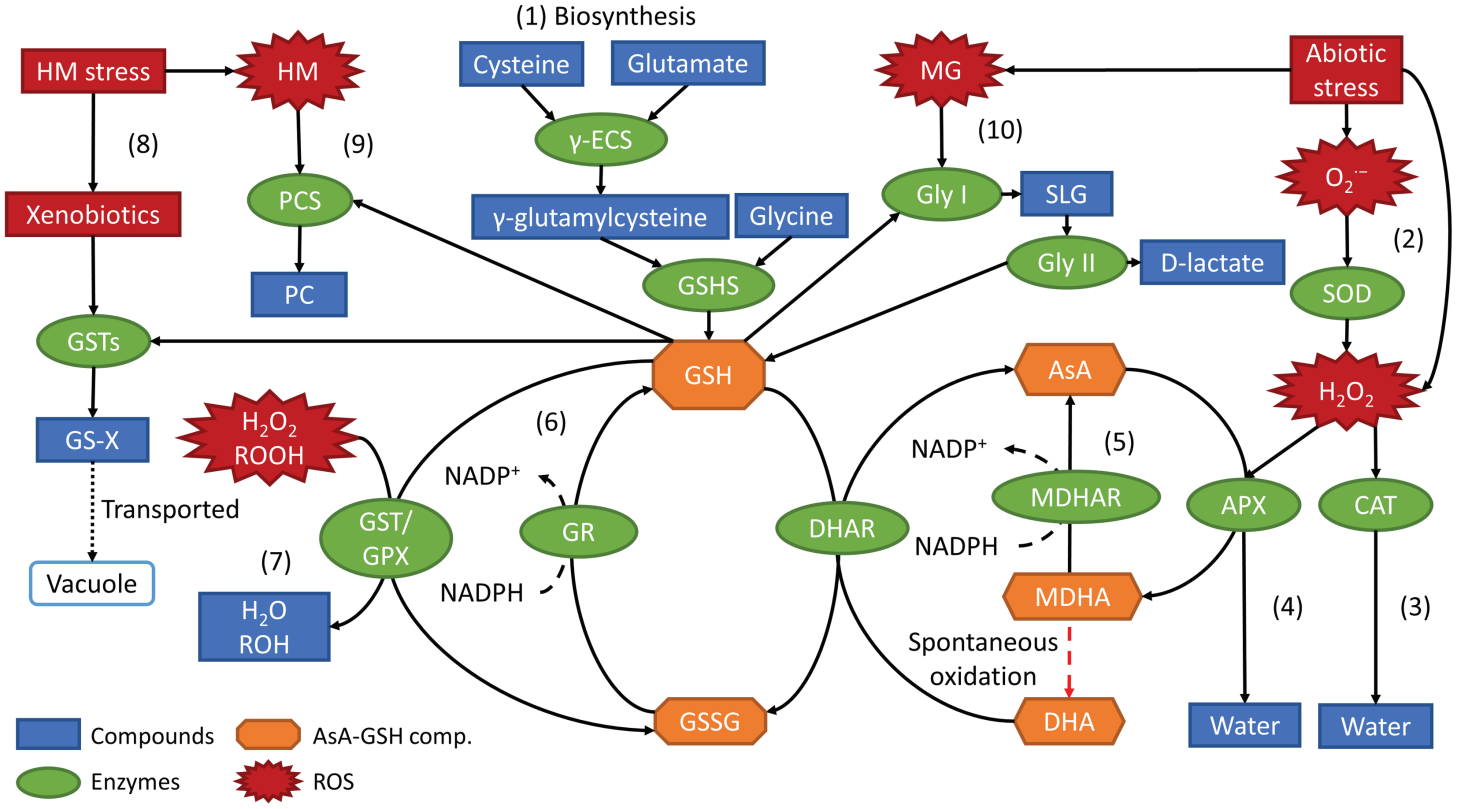
Overview of biosynthesis and roles of GSH along with its metabolism-related enzymes in response to abiotic stresses.

Aside from maintaining the cellular redox homeostasis and its oxidized form GSSG, GSH is also a key component in the ascorbate-glutathione (AsA-GSH) and glyoxalase cycle. GSH removes the excess H_2_O_2_, a stable form of ROS, and reduces MG levels in response to environmental stresses (Cao et al., 2017). Furthermore, GSH also participates in the modulation of gene expression that activates redox-sensitive transcriptional factors and enzymatic activities by regulating the redox state of plants. Besides that, GSH detoxifies xenobiotics, catalyzed by glutathione S-transferase (GST), and sequestration of heavy metals into vacuoles through the formation of phytochelatins (PC). Figure 3 shows an overview of the roles of glutathione and its metabolism-related enzymes in response to abiotic stresses.

From Figure 3, a process labelled (1) shows the biosynthesis of GSH where GSH is synthesized from its constituent amino acids, cysteine, and glutamate, through a two-step ATP-dependent reaction catalyzed by the enzymes γ-ECS and GSHS. As a result of abiotic stresses, ROS species (colored in red), such as $O_{2}^{\cdot -}$, hydroperoxides (ROOH), H_2_O_2_, and MG is typically produced. Process (2) in the right top corner of Figure 3 shows the unstable $O_{2}^{\cdot -}$ anion produced is converted into H_2_O_2_, a stable form of ROS, by an antioxidant enzyme named superoxide dismutase (SOD). Next, H_2_O_2_ converted from $O_{2}^{\cdot -}$ or produced by abiotic stresses, can be converted into water by two pathways depending on the cell compartment, which include, (3) the direct pathway catalyzed by the antioxidant enzyme catalase (CAT); and (4) the AsA-GSH pathway catalyzed by ascorbate peroxidase (APX) at the expense of ascorbic acid (AsA). Other than water, APX catalyzes the oxidation of AsA into monodehydroascorbate (MDHA) and dehydroascorbate (DHA). Process (6) then shows reduction of MDHA and DHA back to AsA, catalyzed by the enzymes monodehydroascorbate reductase (MDHAR) and dehydroascorbate reductase (DHAR), respectively. The formation of DHA happens through spontaneous oxidation from MDHA. At the same time, the reduction of MDHA to AsA utilizes nicotinamide adenine dinucleotide phosphate (NADPH) as an electron donor giving up its electron to be converted into NADP+. Similarly, using NADPH as an electron donor in the process (6), the oxidized form of glutathione, GSSG, is reduced to glutathione, GSH, by the enzyme glutathione reductase (GR). In process (7), H_2_O_2_, ROOH and even lipid peroxides are reduced to either water (H_2_O) or alcohols (ROH) by the enzymes GPX and GST (Cao et al., 2017; Tamaki et al., 2021).

Aside from direct quenching of ROS in the AsA-GSH cycle, there are two other possible pathways specifically for mitigating against heavy metal (HM) stresses, which include processes such as (8) the conjugation of HM-induced xenobiotics by GST and (9) the HM chelation by PC. In process (8), the enzyme GST catalyzes the conjugation of GSH with xenobiotics that include hydrophobic, electrophilic, and cytotoxic substrates to form the conjugate, GS-X (X represents any type of xenobiotics). GS-X is then transported to the vacuoles for further degradation, a crucial response in mitigating HM-induced stress (Hernández et al., 2015; Hasanuzzaman et al., 2017). In process (9), GSH acts as the precursor that allows HMs to bind to their thiol (-SH) group to form PC, catalyzed by the enzyme phytochelatin synthase (PCS). Furthermore, in the defense against stresses, especially salinity stress, MG is produced. MGs are ubiquitous in plants under stress and can be increasingly cytotoxic to plants at higher concentrations, which therefore needs to be detoxified (Hoque et al., 2016). MGs are degraded through the glyoxalase pathway as shown in process (10), which consists of two GSH-dependent enzymes, glyoxalase I (Gly I) and glyoxalase II (Gly II). Gly I converts GSH and MG to S-D-lactoylglutathione (SLG). In contrast, Gly II converts SLG into D-lactate and back to GSH, maintaining the redox homeostasis (Cao et al., 2017). Succinctly, GSH plays a central and vital role in the antioxidant defense system of plants, protecting plants against abiotic stresses through the metabolisms as mentioned above and pathways.

## Roles of Exogenous Gsh in Stressed Plants

**Figure 4:**
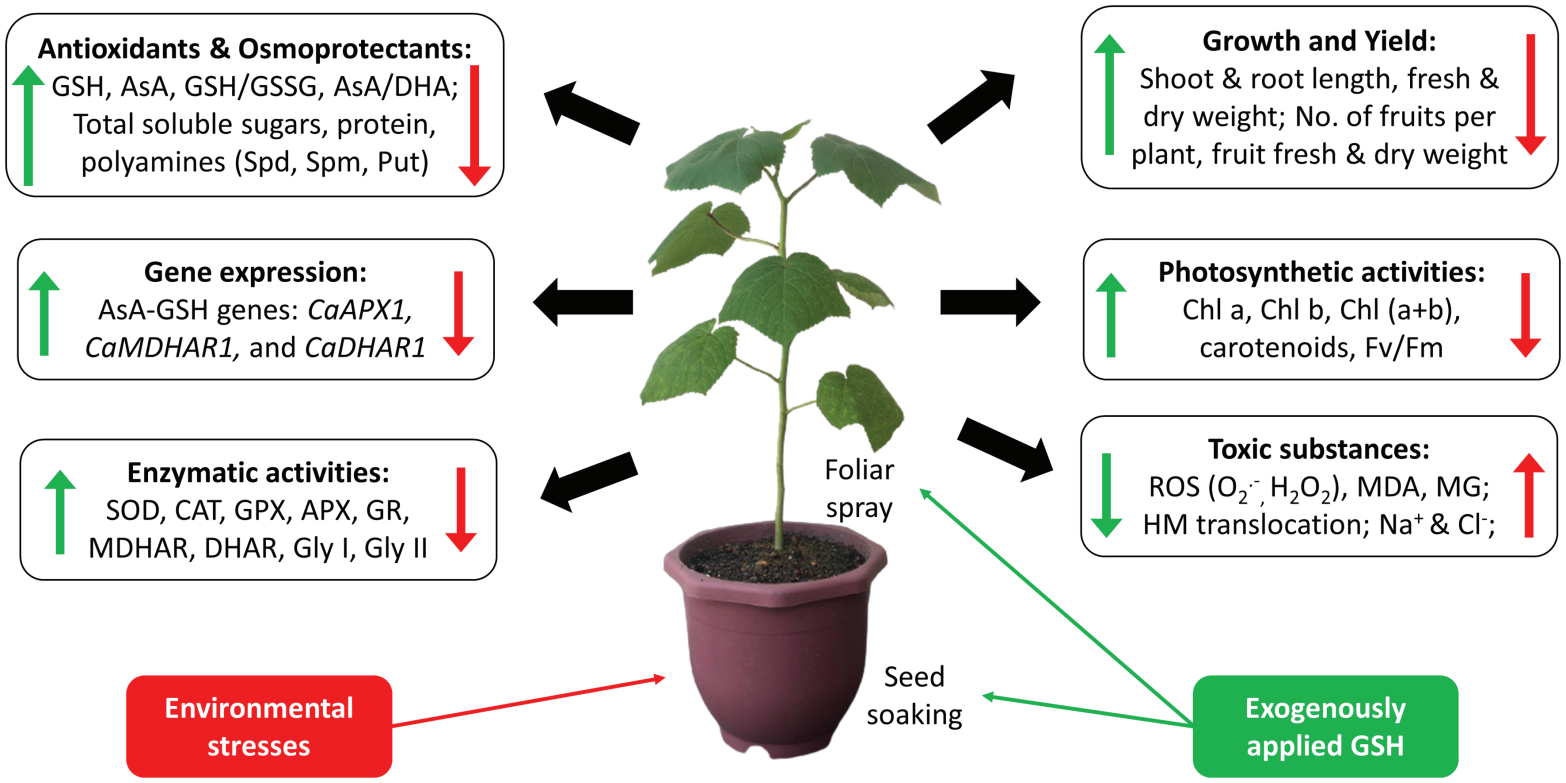
Key effects of individual exogenous application of GSH on plants in response to environmental stresses. Red arrows indicate the effects of environmental stresses in plants; green arrows indicate effects of exogenous application of GSH in response to the environmental stresses.

Exogenously applied GSH is readily taken up and transported into various cellular compartments of plants. The uptake of exogenous GSH usually induces a series of biochemical and physiological processes, including the regulation of abiotic stress tolerance (Cao et al., 2017). Upon understanding the roles of GSH and its metabolism-related enzymes, this section elaborates the role of exogenous GSH in mitigating specific types of stresses, including abiotic stresses such as metal toxicity, salinity, temperature extremities, drought, flood, and even biotic stresses particularly bacterial infection. Figure 4 below shows the critical effects of individual exogenous application of GSH on plants in response to environmental stresses. The key results of recent studies from the year 2017 to 2021 on the individual exogenous application of GSH, paired with its dosage and method of supplementation on various plants against different types of stresses, are summarized in Supplementary Table S1.

### Heavy Metal Toxicity

Heavy metal (HM) contamination in soil has been an increasing concern following the rapid industrialization and intensification of agricultural land use. HMs and metalloids such as cadmium (Cd), lead (Pb), and arsenic (As), among others, have been often to be found in soils, adversely affecting the vegetative growth and yield of various plant crops (Hasanuzzaman et al., 2017; Srivastava et al., 2017). Generally, the uptake of HMs from the contaminated soil by the plants will result in an overproduction of ROS, causing oxidative stress and damage to the plants. In response, GSH, of the antioxidant defense system, participates in the detoxification of toxic HMs through three possible pathways which include, direct scavenging of ROS in the AsA-GSH cycle, HM chelation through the synthesis of phytochelatins (PCs), and conjugation of HMs catalyzed by glutathione S-transferase (GST; Bela et al., 2017b; Cao et al., 2017).

The significance of GSH in the detoxification of HMs was demonstrated in a study performed in upland cotton (*Gossypium hirsutum* L.) plants under lead (Pb) stress (Khan et al., 2016). The exposure of Pb was found to cause an increase in malondialdehyde (MDA, lipid peroxidation marker) and H_2_O_2_ contents (Babenko et al., 2017). The expansion of these oxidative stress markers triggered an increase in antioxidant enzymatic activity of SOD, peroxidase (POD) and GR, but a decline in CAT and APX, possibly due to the limitation of the ROS quenching capacity. The intervention with pre-treatment of GSH was found to cause an increase in the activities of all the antioxidant enzymes and resulted in reduced MDA and H_2_O_2_, alleviating the Pb-induced stress and further increasing the length and width of cotton leaves (Khan et al., 2016). To ensure the sustainability of agricultural food supply, researchers have focused on assessing the effects of exogenous GSH on various food crops such as wheat, brinjal, rice and have demonstrated the successful alleviation of HM stresses such as Cd, Pb, As, and even mercury (Hg; Kim et al., 2017; Jung et al., 2019; Alamri et al., 2021; Li et al., 2021). The treatment of GSH results in increased enzymatic activities such as SOD, CAT, GPX, GR, MDHAR, and DHAR, which leads to an increase in endogenous AsA and GSH levels, along with increased ratios of AsA/DHA and GSH/GSSG, thus reducing the level of overproduced ROS species (Jung et al., 2019; Alamri et al., 2021; Li et al., 2021). On top of enzymatic activities, transcriptomic analysis on the expression levels of GSH and PCs biosynthesis gene, which include *IIγ-ECS, IIGS, IIPCS*, were found to be upregulated after 24-h treatment of GSH against Pb induced stress on leaves of the plant known as *Iris lactea* var. *chinensis* (Yuan et al., 2018).

Besides increasing enzymatic activities and upregulation of GSH biosynthesis gene expressions, exogenous GSH plays a significant role in regulating photosynthetic activities (Wang et al., 2011; Khan et al., 2016; Li et al., 2021). Wang et al. (2021) carried out a greenhouse hydroponic experiment using two different barley genotypes, Cd-sensitive Dong 17 and Cd-tolerant Weisuobuzhi, to investigate the effects of exogenous GSH on their photosynthetic performances under Cd stress. It was revealed that the treatment of GSH significantly increased both genotypes, especially Cd-sensitive Dong 17, with a great increase in parameters such as the net photosynthetic rate, stomatal conductance, transpiration rate and photochemical efficiency (F_v_/F_m_) by 130.2, 68.9, and 52.4%, respectively, when compared to the control plant (Wang et al., 2011). Furthermore, the roles of exogenous GSH in improving the photosynthetic performance of plants were reaffirmed through a significant upsurge detection in photosynthetic pigments level such as chlorophyll a (Chl a) and chlorophyll b (Chl b) in GSH-treated wheat seedlings subjected to Cd stress (Li et al., 2021).

The prominent use of GSH in reducing HM uptake and transport is deemed promising and is being explored extensively by researchers in very recent studies (Fang et al., 2020; Li et al., 2021). The uptake and subcellular distribution of Cd in Italian ryegrass (*Lolium multiflorum)* under Cd stress and GSH treatment was reported by Fang et al. (2020) recently. The results showed an increase in Cd accumulation in roots but a decrease in shoots, proving a decrease in Cd translocation from roots to shoots with the application of GSH. Besides that, the study found that most Cd was present in the soluble fraction, mainly in vacuoles, of the leaves under Cd stress only. With the addition of GSH treatment, the Cd concentration in the soluble fraction increased. In contrast, the other subcellular fraction of leaves decreased significantly, suggesting that the Cd ions were compartmentalized mainly in the vacuoles. This finding proves that the supplementation of GSH improves the vacuolar compartmentalization and is crucial for the detoxification of Cd. (Fang et al., 2020). Through transcriptomic analysis, the expression levels of the Cd transporter, including *TaNramp1, TaNramp5, TaHMA2, TaLCT1* on the roots and shoots of wheat plants, were found to be successfully suppressed by the exogenously applied GSH (Li et al., 2021).

Apart from the uptake and distribution of HM, metabolomics, which is the study of the identification, quantification, and distribution of metabolites, such as sugars, alcohols, amino acids, organic acids, and polyamines, provides a better understanding of the plant’s responses to any interference, including HM stresses (Ghatak et al., 2018). In the metabolome study of maize leaves under Cd stress, the tricarboxylic acid (TCA) cycle-related metabolites such as malic acid and maleic acid were found to be decreased in leaves, possibly due to the nature of its enzyme known as fumarate hydratase, that was reported to be particularly sensitive to ROS (Lushchak, 2011; Wang et al., 2021). With the addition of GSH, the trend of the TCA cycle-related metabolites was reversed, where malic and maleic acid levels were detected to increase in leaves, implying that the exogenous GSH successfully alleviates the oxidative stress induced by Cd toxicity on the TCA cycle (Wang et al., 2021). Besides TCA cycle-related metabolites, sugar contents such as maltose, fructose, heptanose, sophorose and sucrose in plants under the treatment of GSH were significantly increased. Notably, the accumulation of soluble sugars such as fructose and sucrose help maintain the osmotic balance of plants under stress, reducing the adverse effects of ROS induced by Cd stress (Li et al., 2017; Ševčíková et al., 2017; Živanović et al., 2020). Furthermore, the addition of GSH increases the endogenous GSH levels, followed by the rise of osmotic and antioxidant moieties such as allo-inositol, ascorbic acid-related and flavonoid-related metabolites, in both leaves and roots. The increase of these metabolites proves the role of exogenous GSH in ameliorating lipid peroxidation and alleviating Cd-induced toxicity on maize plants (Wang et al., 2021).

In short, it can be concluded that the roles of exogenous application of GSH in mitigating HM toxicity are mainly related to direct scavenging of ROS, which is regulated by the antioxidant enzymatic activities, and up-regulation of stress response gene expression; increasing photosynthetic activities and performances; decreasing HM translocation and regulating the homeostasis of ions; and increasing osmotic and antioxidant metabolites.

### Salinity Stress

Salinity stress is considered one of the most severe constraints to agricultural production globally, affecting close to 20% of the irrigated land and forecasted to affect up to 50% of the land by the year 2050, becoming a major concern for global food security (Hussain et al., 2016; Cao et al., 2017; Hasanuzzaman et al., 2017). Salt tolerant mechanisms are quite complex as it involves both osmotic adjustment and toxic ion compartmentation (Khattab, 2007). Plants exposed to high salinity stresses usually suffers from both osmotic and ionic stresses. Osmotic stress is developed due to the significant reduction in water uptake as an effect of increased salt concentration. It is often followed by the formation of ionic stress on plant, mainly due to the excessive accumulation and absorption of toxic ions in plants (Teh et al., 2015). On top of that, just like any other stress, salinity stress also results in overproduction of ROS, inducing secondary oxidative stresses on plants (Hussain et al., 2016).

The roles of endogenous GSH in maintaining salt tolerance in plants were previously demonstrated by comparing two rice cultivars, salt-tolerant BRRI dhan54 and salt-resistant rice BRRI dhan49 (Hasanuzzaman et al., 2014). Upon salt stress conditions, the salt-tolerant cultivar., BRRI dhan54, showed a higher GSH content, a higher GSH/GSSG ratio and higher GST and GPX activities, leading to a significant reduction in H_2_O_2_ and MDA as compared to the salt-sensitive BRRI dhan49, proving the significance of GSH in alleviating the salt-induced stress on plants (Hasanuzzaman et al., 2014).

Similarly, exogenous application of GSH was found to positively correlate to the increase in antioxidant enzymatic and photosynthetic activities in salt-stressed plants (Akram et al., 2017; Ibrahim et al., 2017). Ibrahim et al. (2017) proved that the supplementation of GSH on both stress-tolerant and sensitive genotypes of cotton seedling (Zhong 9806 and Zhongmian 41) had successfully alleviated salinity stress especially in the salt stress-sensitive genotype, Zhongmian 41. This is due to the enhanced activities of antioxidant enzymes such as SOD, POD, CAT and APX, together with the increase in photosynthetic pigments such as chlorophyll a, chlorophyll b, carotenoids, and total pigments (Ibrahim et al., 2017). In response to ionic and osmotic stresses induced by salinity stress, parameters such as ionic homeostasis and osmoprotectant contents are crucial and were evaluated in recent studies (Al-Elwany et al., 2020). Chili peppers *Capsicum frutescence* (L.) is one of the most popular vegetable crops cultivated in Egypt, have been reported to particularly sensitive to salinity stresses which deemed as the key factor leading to a significant drop in crop production level (Al-Elwany et al., 2020; Kopta et al., 2020). In view of the severity, Al-Elwany et al. (2020) conducted a pot experiment of the chili peppers under salinity stress of 6.74 dS m^−1^ and exogenous GSH treatment *via* a foliar spray of 0.8 mM being applied in order to evaluate for its potential efficacy. The findings demonstrated the GSH treated plants under salinity stress showed improvement in plant growth and yield, especially through the staggering increment in the number of fruits per plant by 235% and plant dry fruits by 258.1%, compared to chili pepper plants under salinity stress. The significant improvement is contributed by the role of GSH in increasing the osmoprotectant contents such as soluble sugars by 30.2%, phenolic content by 25%, capsaicin content by 25.6%, and antioxidant contents such as AsA by 18.1% and GSH by 55%. In response to the increased content of Na^+^ and Cl^−^ induced by salinity stress, the addition of GSH was also responsible for regulating ionic homeostasis, reducing Na^+^ and Cl^−^ content while increasing macronutrients like K^+^, Ca^2+^ and Mg^2+^ (Al-Elwany et al., 2020).

In response to salinity stress, the recent completed studies on tomato (*Solanum lycopersicum* L. cv. Zhongshu No. 4) seedlings in China concluded the roles of exogenous GSH is mainly through the modulation of the ion homeostasis and polyamine metabolism pathways (Xu et al., 2000; Zhou et al., 2019). Under 100 mM of salt (NaCl) stress, the tomato seedlings resulted in retardation in seedling growth, caused by the imbalance of ionic homeostasis due to the excessive accumulation and uptake in Na^+^ and Cl^−^_._ Meanwhile, the supplementation of GSH successfully reversed the Na^+^ and Cl^−^ back to normal levels, thus reducing the ion-selective transport coefficient of Na^+^ as compared to other ions like K, Ca, and Mg (S_Na, K,_ S_Na, Ca_ and S_Na, Mg_). In response to osmotic stress induced by the salt stress, tomato seedlings significantly increased the activities of polyamines (PAs) such as spermidine (Spd), spermine (Spm), and putrescine (Put), which contributes to the osmotic adjustment to maintain cell turgidity. However, the addition of exogenous GSH reduced activities of the PA synthesis-enzymes like arginine decarboxylase (ADC), S-adenosylmethionine decarboxylase (SAMDC), and diamine oxidase (DAO), while increased the activities of polyamine oxidase (PAO) and ornithine decarboxylase (ODC), resulting in reduced levels of PAs such as Spd, Spm and Put. This study proved the interaction of GSH and PAs is crucial in a plant’s adaptive response to ionic and osmotic stress induced by salinity stress (Zhou et al., 2019).

In summary, in response to osmotic and ionic stresses induced by salinity stress, exogenous application of GSH plays vital roles, including regulating the antioxidant enzymatic activities, photosynthetic activities, homeostasis of ions, osmoprotectant values and polyamine activities.

### Temperature Extremities – Cold Stress

As a direct effect of climate change, temperature extremities be it high or low, have become significant environmental factors limiting agricultural plant production (Hasanuzzaman et al., 2017). Interestingly, the recent years of studies conducted are demonstrating a common phenomenon, whereas cold stress has somehow emerged as the prominent focus instead of heat stress which results in a big contrast against previous years’ of research focus. In fact, based on our knowledge, there is a null scientific research article in the area of heat stress study from 2018 to 2021 and drawing our attention to focus on recent research progress of GSH on cold stress. Cold stress is oxidative stress induced by chilling or freezing temperatures that adversely affect plant growth and development (Cao et al., 2017). Like hot stress, cold stress induces an excessive generation of ROS, leading to attenuation of the plant’s ROS scavenging ability, disrupting ROS homeostasis. This disruption may lead to lipid peroxidation, protein damage, cell structure destruction, and chilling injury, especially in postharvest fruits or vegetables, which is a specific symptom of cold stress (Yao et al., 2021).

Previous studies have shown a strong correlation between endogenous GSH content and cold tolerance of various plant crops such as maize, wheat, chickpeas, and mung bean (Hasanuzzaman et al., 2017). Particularly, cold priming in *Jatropha curcas* seedlings, a potential source of biodiesel, resulted in enhanced activities of antioxidant enzymes such as SOD, APX, CAT, GR, which correlated to higher content of AsA, GSH and proline, proving the significance of GSH in mitigating cold stress (Li et al., 2013). The AsA-GSH was also proven to be of great importance in enhancing chilling tolerance in banana plants by increasing enzymes like MDHAR and DHAR in the AsA-GSH cycle (Liu et al., 2019).

To decipher the role of GSH in gene expressions under cold stress, transcriptome analysis of the agronomically important crop – rice plants under cold stress treated with exogenous GSH was performed and resulted in upregulation of gene expression involved in transcription like *DREB* and *ERF*, hormone biosynthesis and signaling like *AOS, LOX, JAZ*, and *SAUR* in the roots (Park et al., 2021). A further study was conducted using transgenic rice (TR) plant that was overexpressed with *OsGS*. This gene that encoded the final enzyme for biosynthesis of GSH and compared its mechanism of GSH in mitigating cold stress against the wild type (WT) of rice. The TR plant overexpressed with *OsGS* under cold stress showed an average increase in endogenous GSH by 1.53-fold, and GSH/GSSG ratio by 3.4-fold, compared to WT of rice plant. The increase in endogenous GSH led to a more substantial decrease in ROS species such as H_2_O_2_ and MDA, alleviating cold stress-induced damage. The alleviation of cold stress enhanced the root growth of TR plants which further increased its plant growth and grain yields, such as increased panicles and spikelets, ultimately improving the cold stress tolerance of TR plants when compared to WT rice plants (Park et al., 2021).

Instead of the conventional studies on growing plants, the effect of exogenous GSH on the postharvest bell pepper fruits (*Capsicum annuum* L.), and the roles of the AsA-GSH cycle in mitigating cold stress was being investigated. The untreated bell pepper fruits resulted in severe plasmolysis, where the chilling injury (CI) index was significantly increased with the increase in time. Interestingly, the bell pepper fruits treated with GSH only showed in mild CI symptoms with no visible plasmolysis and well-maintained cellular integrity. The maintenance of ultrastructure is contributed to the role of GSH in increasing the enzymatic activities of APX, GR, and MDHAR of the AsA-GSH cycle that leads to reduced levels of ROS, such as O_2_^−^, H_2_O_2_, and MDA. The importance of the AsA-GSH cycle in alleviating cold stress was further reinforced with the transcriptomic analysis on the AsA-GSH cycles genes, which shows upregulation of genes such as *CaAPX1, CaGR2, CaMDHAR1*, and *CaDHAR1* (Yao et al., 2021). Hence, it can be concluded that the exogenous GSH indeed leads to the increase in endogenous GSH, key to regulation of the AsA-GSH cycle, which oversees detoxification of ROS species, alleviating any cold stress-induced damage.

### Drought

Following the increasing climate warming, drought or water scarcity has become one of the biggest concerns to global agricultural productivity, especially in warmer and arid regions like Africa (Sohag et al., 2020; Seleiman et al., 2021). Drought is considered one of the most severe environmental stresses due to unpredictable factors such as fluctuating temperature dynamics, light intensity, and undependable rainfall (Seleiman et al., 2021). Drought generally causes an osmotic imbalance in plants, which leads to interruption of stomatal conductance, electron transport rate, carboxylation efficiency and photosynthesis. The impairment of these physiological aspects leads to excess ROS generation, when exposed to prolonged or extreme levels of drought stress (Hasanuzzaman et al., 2017; Sohag et al., 2020). The overproduced ROS may lead to lipid peroxidation, generating MDA, a critical oxidative stress marker, especially in drought stresses (Kong et al., 2016).

The previous extensive studies on drought-stress resistant plant species revealed the significant roles of both AsA and GSH in regulating of metabolic processes in response and adaptation of the plant to drought stress (Hasanuzzaman et al., 2012; Çevik and Ünyayar, 2015; Cao et al., 2017). Drought has become a recurring problem in warm, arid, semi-arid regions, limiting the food crop productivity that includes chickpea (*Cicer arietinum* L.), the third most important pulse crop in global production (15.42%; Sen et al., 2012; Merga, 2019). Studies were carried out to understand the mechanism of chickpea plants in frequent drought-stressed regions like Turkey (Çevik and Ünyayar, 2015; Guneri et al., 2019). Çevik and Ünyayar (2015) demonstrated that the drought-tolerant chickpea, *Cicer reticulatum* Ladiz. AWC611 showed higher AsA and GSH contents than the drought susceptible species *Cicer arietinum* L. ILC8617. The results also showed higher enzymes activity involved in ROS scavengings such as SOD and APX (Çevik and Ünyayar, 2015). Furthermore, lipoxygenase (LOX) activity, the enzyme that catalyzes lipid peroxidation, was studied and found to increase significantly when plants were subjected to drought stress (Nahar et al., 2015; Babenko et al., 2017). Besides enzymatic activities, relative water content (RWC) is a crucial parameter in assessing the physiological water content of the plants. RWC resulted in a substantial decrease in response to drought stresses, especially in the leaves (Nahar et al., 2015).

Similarly, in a warm region like Bangladesh, drought stress poses a severe threat to agricultural production in the northwest part of the country (Habiba et al., 2012). Hence, in efforts to improve rice (*Oryza sativa* L.) plant tolerance under drought stress in Bangladesh, Sohag et al. (2020) investigated the roles and mechanisms of exogenous GSH-mediated drought stress tolerance using drought susceptible rice (cv. BRRI dhan29). The addition of 0.2 mM of GSH for 72 h on the drought-stressed plant showed significant improvement in growth characteristics such as longer roots, a greater weight of both shoots and roots. The alleviation of drought stress is proven through the decrease in ROS species and MDA content due to the increased enzymatic activity of CAT, APX, and POD, which further increased the content of antioxidants like AsA and carotenoids. Besides antioxidants, leaf osmotic adjustment through the regulation of solutes such as proline (Pro) and total soluble sugars (TSS) is essential to increase the tolerance of plants under drought stress. Interestingly, pre-treatment of GSH resulted in a higher RWC and TSS, but a decreased Pro content, indicating that the GSH has contributed to the reduction of osmotic stress (Nahar et al., 2015; Sohag et al., 2020). Hence, exogenous application of GSH successfully ameliorated the drought-induced osmotic and oxidative stress through enhancing antioxidant systems and regulation of leaf osmotic solutes.

### Flood/Submergence Stress

As opposed to drought, flood is the occurrence of excess water that causes a composite and complex stress, known as submergence (Sub) stress of plants (Fukao et al., 2019; Siddiqui et al., 2021). The Sub stress is deemed as one of the three worst environmental stresses along with salinity and drought stress. It is known adversely affecting the productivity and even survival of the agricultural ecosystem, especially in rain-fed lowlands of the world (Siddiqui et al., 2021). For instance, Bangladesh is one of the most flood-prone countries, often affected by torrential rain, tidal surges and glacier melt of the Himalaya region (Dewan, 2015). Overexposure of plants to Sub stress typically results in hypoxia, shortage of oxygen, or anoxia, a total absence of oxygen, which ultimately leads to tissue damage or plant death in the worst case (Loreti and Striker, 2020). Like any other abiotic stress, Sub-induced stress results in restriction of photosynthetic capacity and overproduction of ROS, which can cause oxidative damage to the plant’s enzyme, protein, and lipids (Siddiqui et al., 2021). The role of exogenous GSH in alleviating abiotic stresses like salinity, drought, and metal toxicity has been extensively studied. Still, its role in relieving Sub stress has yet to be explored. Therefore, Siddiqui et al. (2021) investigated the effects of exogenous application GSH on two high-yielding rice (*Oryza sativa*) varieties, namely BRRI dhan29 and BRRI dhan52 (Sub-tolerant), in mitigating Sub-stress in Bangladesh.

For instance, the project was designed as such- on the 20th day of planting, seedlings were subjected to either 1 mM or 2 mM of GSH treatment *via* foliar spray on every second day for 2 weeks. On the 35th day of planting, seedlings from both rice cultivars were fully submerged in a pond for 14 days. Upon 24-h recovery, the results for both cultivators exposed to Sub-stress showed chlorosis, wilting of older leaves and growth retardation that includes shorter shoot height, less fresh and dry weight of shoots. The adverse effects are best explained by the sharp reduction in photosynthetic pigments and reduction in enzyme activities that correlate to the increase in ROS species like MDA and H_2_O_2_. The treatment groups with GSH, especially of 2 mM concentration, successfully reversed the adverse effects, resulting in the tallest shoot height, and the greatest fresh and dry weight of shoots. The exogenous GSH increased the chlorophyll pigments, Chl a, Chl b, total chlorophyll (Chl a + b), carotenoids, soluble proteins contents, along with the increase in enzymatic activities such as SOD, CAT, POD, APX, GPX and GST, which resulted in the reduction of the overproduced ROS species, alleviating Sub-stress in rice plants. The cultivator dhan 52 scored higher stress tolerance indices (STI) than dhan29, validating that dhan 52 is a Sub-tolerant rice cultivar. Interestingly, the exogenous application of GSH in two different cultivars showed slight differences in the enzymatic defense, where a more remarkable recovery was achieved for activities of SOD, CAT, POD for dhan 29, and APX, GPX, GST in dhan 52. Hence, demonstrating exogenous GSH was effective in amelioration of the oxidative damage caused by Sub-stress and further improves Sub-stress tolerance in rice plants. Perhaps in future, a further transcriptomic analysis on the gene expressions and analysis on molecular levels are recommended to thoroughly understand the mechanisms of GSH-mediated Sub-stress tolerance as it might shed light on how it might can be utilized for various important crops (Siddiqui et al., 2021).

### Biotic Stress – Bacterial Infection

Biotic stresses are generally caused by living organisms such as bacteria, fungi, nematodes, viruses, and insects. These living organisms, also known as pathogens and pests, are directly accountable for infecting plants with diseases, depriving them with nutrients and in worst case plant death (Kovalchuk, 2016; Gull et al., 2019). Among the pathogens, fungi and bacteria may induce symptoms like vascular wilts, leaf spots, and canker (death of tissues), infecting various parts of the plant (Moustafa-Farag et al., 2020). Nematodes are multicellular insects that cause soil-borne diseases, while viruses can cause both local and systemic damage to the plant, leading to symptoms like chlorosis, stunted growth and wilting (Osman et al., 2020). Insects, on the other hand, often act as vectors of most bacteria and viruses either through feeding or laying eggs on the plants, directly depriving plants of the nutrients (Moustafa-Farag et al., 2020; Iqbal et al., 2021). In defense to the biotic stresses, plants have a complex array of defense mechanism (Saijo and Loo, 2020). Their passive first line of defense includes physical barriers such as trichomes, cuticles, and wax, that averts pathogens and insects, followed by their capability of producing chemical compounds that defends themselves from pathogen infections (Zaynab et al., 2018). On top of that, when plants detect a biotic stress condition, it triggers a defense by two levels of pathogen recognition, activating the regulatory or transcriptional system, generating the appropriate defense response (Moustafa-Farag et al., 2020; Iqbal et al., 2021).

Interestingly, by comparison, the mitigation effects of exogenous GSH in abiotic stresses is far stretched and well documented versus in the biotic stresses use. For example, the rather recent study investigated the exogenous application of GSH on chili (*Capsicum annum* L.) seedlings in mitigating *Xanthomonas campestris*, bacterial spot disease, and proved significant roles and effects of exogenous GSH in mitigating biotic stresses. *X. campestris* is one of the most severe bacterial diseases contributing to the reduction in plant production globally (Ramzan et al., 2021). Infected plants usually result in spotting on leaves and fruits, accompanied by decreased in photosynthetic activities (Horváth et al., 2015). Photosynthesis remains one of the most pivotal mechanisms in plants, and its regulation is of utmost importance in response to biotic stresses (Ochs, 2021). In view of the importance of photosynthesis, chlorophyll fluorescence parameters such as maximum quantum yield of PSII (F_v_/F_m_), photosystem efficiency (ΦPSII), photochemical quenching (qP), photon energy absorbed in PSII used for photosynthesis (P%) and dissipated *via* thermal energy (D%) among others are utilized in experiments to investigate photosynthetic activities, a correlation between host plant-stress factors and even pathogen location (Ramzan et al., 2021). The chili seeds inoculated with *X. campestris* showed varying spotting symptoms on the leaves and lower plant dry weight due to decreased F_v_/F_m_, qP and relative electron transport rates (ETR). Exogenous application of GSH before inoculation of the seeds successfully alleviated the biotic stress by increasing the photosynthetic pigments, improved chlorophyll fluorescence parameters like F_v_/F_m_, ΦPSII, qP, P% and D%. In addition, the exogenous GSH reduced the accumulation of ROS such as H_2_O_2_ and MDA by increasing antioxidant enzymatic activities like SOD, POD, and CAT (Ramzan et al., 2021). Hence, exogenous applications of GSH have ameliorated oxidative damage, enhanced biotic stress tolerance, and further improved the growth and development of chili plants.

## Sequenced or Combined Application of Gsh and Bioactive(S)

**Figure 5:**
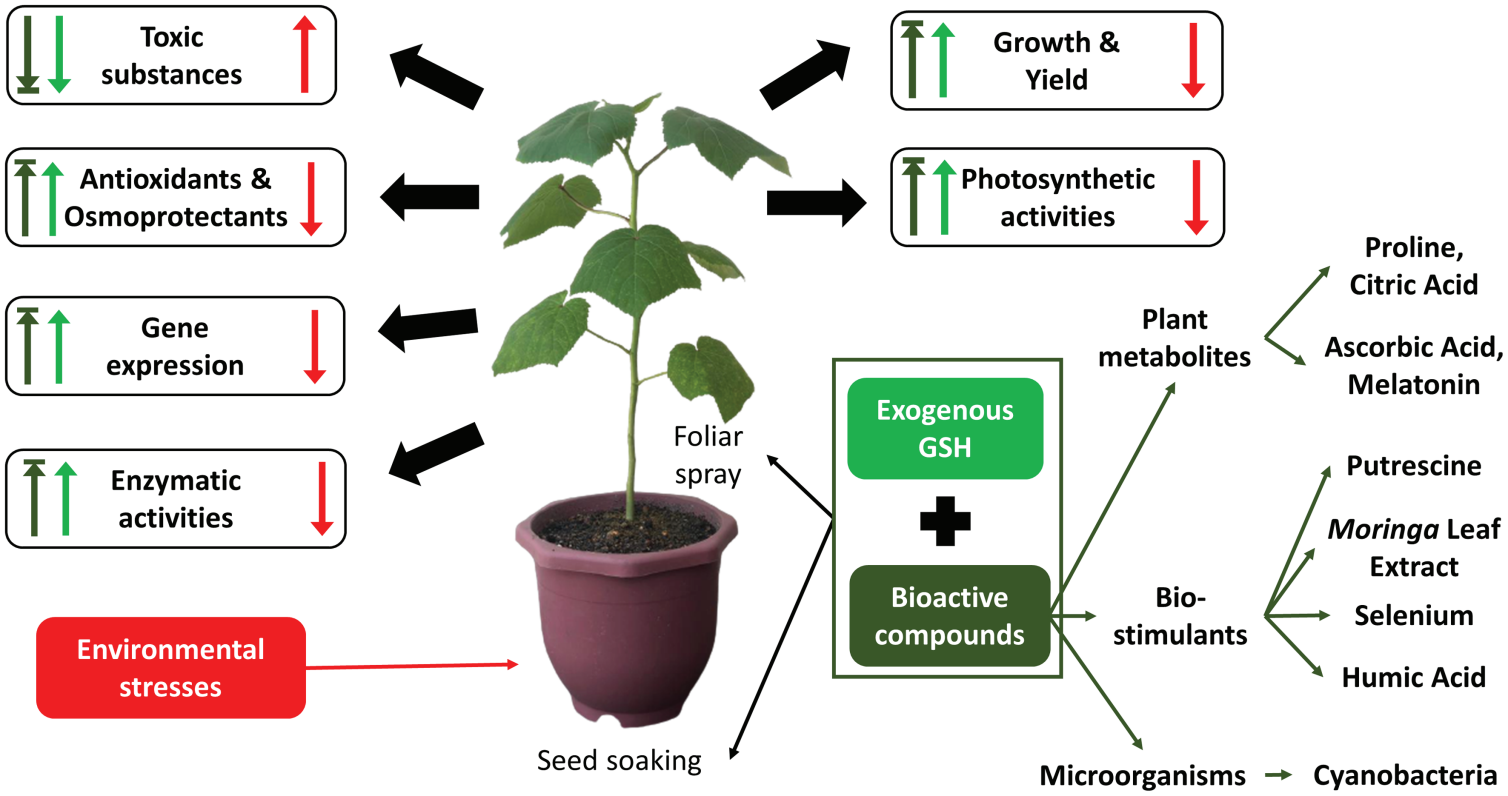
Summarized key effects of combined application of exogenous GSH and different bioactive compounds on plants in response to environmental stresses studied in year 2018–2021. Red arrows indicate the effects of environmental stresses in plants; light green arrows indicate effects of individual exogenous application of GSH; dark green arrows indicate effects of combined application of exogenous application of GSH and other bioactive compounds in response to the environmental stresses.

From section 5, it was clear that the single exogenous application of GSH can effectively mitigate the different types of environmental stresses. However, the improvement of plant growth and yield contributed by the exogenous application of GSH may be limited. Hence, there has been an increasing trend of recent studies investigating the sequenced or combined application of GSH with different bioactive(s), which can be categorized into three main types: plant metabolites (primary & secondary) and biostimulants. The section below elaborates the properties and specific roles of the different bioactive compounds in the sequenced or combined application with GSH against various stresses, according to the categorized bioactive compounds. Figure 5 below shows summarized key effects of both individual exogenous application of GSH and combined GSH and bioactive compounds on plants in response to environmental stresses. The key results of recent studies from the year 2017 to 2021 on the sequenced or combined application of GSH with other bioactive compounds, together with their targeted stress, dosage and method of application are summarized in Supplementary Table S2.

### Plant Metabolites

Plant metabolites are compounds or substances synthesized by complex physical and chemical events, including respiration, photosynthesis, and degradation (Bowsher, 2019). Previous studies have successfully demonstrated the significance and roles of plant metabolites such as GSH, proline (Pro) and ascorbic acid (AsA) in enhancing plant tolerance against various types of environmental stresses (Rady and Hemida, 2016; Semida et al., 2018; Godoy et al., 2021). It is known that plants, fungi and microorganisms produce over 200,000 metabolites and can be categorized into two main groups, primary and secondary metabolites.

Primary metabolites are synthesized by all living organisms and are involved in vital processes such as growth and development, along with the synthesis of essential proteins, lipids, and carbohydrates (Godoy et al., 2021). Examples of primary metabolites include proline (Pro), GSH, citric acid (CA) and tryptophan (TRP). Among them, extensive studies have been performed on the exogenous application of Pro and CA in efforts to mitigate abiotic stresses that include salinity, metal toxicity and heat stress, which will be further elaborated in subsections 4.1.1 and 4.1.2, respectively (Sun and Hong, 2011; Abdelhamid et al., 2013; Hu et al., 2016).

On the other hand, secondary metabolites are synthesized in different cellular compartments and function as signaling molecules and defense against environmental stresses (Godoy et al., 2021). Some common secondary metabolites include sorbitol, lipoic acid (LA), AsA, glycine betaine (GB), alpha-tocopherol (α-Toc), and melatonin (MT). Among them, the effects of AsA and MT in mitigating abiotic stresses against drought, salinity and metal toxicity has been well documented and will be elaborated in subsections 4.1.3 and 4.1.4, respectively (Liang et al., 2018; Zhang et al., 2019; Ahmad et al., 2021; Munir et al., 2021).

#### Citric Acid

CA is the initial intermediate of the TCA cycle. It plays a vital role as a mild antioxidant and metal chelation agent that interacts effectively with HMs to facilitate their immobilization, which alleviates the HM-induced stress on plants (Godoy et al., 2021; Zeng et al., 2021). Recently, Zeng et al. (2021) investigated the effects of combined application of GSH and CA on castor bean (*Ricinus communis* L.) plants and demonstrated successful results in alleviating Pb-induced stress. The synergistic effects of GSH and CA are shown through the improved growth parameters such as length, dry weight of shoots and roots, number of leaves per plant and leaf area in Pb-stressed plants. The enhanced biomass and growth traits are correlated to the increase in water use efficiency (WUE), stomatal conductance, transpiration rate and photosynthetic performance. The amelioration of Pb-induced stress was also evident as enzymatic activities such as SOD, POD, CAT, APX were significantly increased, which led to the significant decrease in the overproduced H_2_O_2_, MDA and electron leakage (EL) in the roots and leaves (Zeng et al., 2021).

#### Proline

Pro is a low-molecular-weight amino acid known as an essential antioxidant that functions as a key ROS scavenger, maintaining the cellular redox of plants (Semida et al., 2018). Besides that, Pro is also known as an osmoprotectant that acts as a molecular chaperone, protecting the protein and membrane integrity (Seleiman et al., 2020; Godoy et al., 2021). Previous studies on the single exogenous application of Pro have successfully alleviated stresses that include salinity and metal toxicity (Singh et al., 2015; Teh et al., 2015). For instance, Teh et al. (2015) reported a significant increase in plant growth parameters when 5 and 10 mM of Pro was exogenously applied to the rice plants under salinity stress. Given the previous successful examples, the combined application of GSH, Pro and AsA, a secondary metabolite, was extensively studied on various plants against abiotic stresses and elaborated in the subsection below.

#### Ascorbic Acid

Ascorbic acid (AsA), known to many as vitamin C, is one of the most potent and prevalent antioxidants in most plant tissues, with almost up to 10 times higher concentrations than GSH (Seleiman et al., 2020). AsA has several critical roles in the plants’ cellular processes, including enzyme cofactor for the phytohormones biosynthesis, cell division regulation, and subsequent growth and development (Rady and Hemida, 2016; Godoy et al., 2021). Given their benefits, several research studies on the sequential combined application of GSH, AsA and Pro were carried out in recent years to investigate the effects of combined antioxidants against abiotic stresses like drought, salinity, and metal toxicity (Semida et al., 2018; El-Beltagi et al., 2020; Seleiman et al., 2020). For instance, Semida et al. (2021) investigated the effects of not just combined but the different sequential applications of GSH, AsA and Pro, on Faba beans (*Vicia faba* L.) in mitigating salinity stress (4.53 dS m^−1^). Interestingly, the sequential combination of AsA-Pro-GSH showed the best growth and highest yield, followed by a single application of GSH, a sequential combination of GSH-Pro-AsA, a single application of AsA and Pro. The successful amelioration of ionic and osmotic stress induced by salinity stress was highly correlated to the best photosynthetic parameters such as chlorophyll content, stomatal conductance, and Fv/Fm, the best improvement in leaf relative water content (RWC), membrane stability index (MSI) and water use efficiency (WUE), the highest content of AsA and GSH (Semida et al., 2021).

#### Melatonin

Melatonin (MT; N-acetyl-5-methoxytryptamine) is a tryptophan derivative that acts as a potent antioxidant, neutralizing the overproduced ROS in stressful conditions, mitigating oxidative-induced damage, and further stimulating the growth and development of plants (Goodarzi et al., 2020; Godoy et al., 2021). Recently, the effects of combined application of GSH and MT was explored on safflower seeds (*Carthamus tinctorius* L.) cv. Arak2811 under 500 μm of zinc (Zn) stress. The combined application of GSH and MT resulted in the best improvement of growth, biomass production and chlorophyll content of Zn-stressed safflower seedlings compared to the single application of GSH or MT. The alleviation of Zn was also proven through the significantly reduced oxidative stress markers like MDA and H_2_O_2_ and increase in antioxidant ratios such as AsA/DHA and GSH/GSSG. As opposed to other studies, the enzymatic activities of SOD, enzymes participating in the AsA-GSH cycle (DHAR, MDHAR and GR), and enzymes of the glyoxalase system (Gly I and Gly II) showed a markedly decrease. This is possibly attributed to the strong antioxidant properties of GSH and MT that performs rapid and direct scavenging of ROS, hence reducing enzymatic activities back to normal (Goodarzi et al., 2020).

### Biostimulants

Biostimulant is defined as any substance, usually of a natural origin, applied to plants to improve abiotic stress tolerance, enhance nutrition efficiency and increase crop quality (du Jardin, 2015). Biostimulants act as a trigger for the natural defenses of plants, stimulating the increase in stress tolerance without leaving adverse effects on the yield and qualities of the crop (Abd El-Mageed et al., 2017; García-García et al., 2020). Biostimulants contain a wide range of bioactive compounds. They can be categorized into 7 main groups: protein hydrolysates and N-containing compounds, botanical extracts, inorganic compounds, humic and fulvic acids, biopolymers, beneficial fungi and bacteria (du Jardin, 2015). Among the seven types of biostimulants, four of them from the groups, protein hydrolysates and N-containing compounds, botanical extracts, inorganic compounds, humic and fulvic acids, were combined with GSH to investigate their combined effects on plants in response to abiotic stresses such as salinity and metal toxicity (Dawood et al., 2020; Elkhatib et al., 2021; Jahan et al., 2021; Rehman et al., 2021). A brief introduction and key results of the combined application of GSH and biostimulants were elaborated in the subsections below.

#### Protein Hydrolysates and N-Containing Compounds – Putrescine

Aside from peptides and amino acid compounds, protein hydrolysates also contain other nitrogenous molecules that include phenols, polyamines, fats, and carbohydrates (Colla et al., 2015). Recently, polyamines, especially putrescine (Put), have been regarded as great plant biostimulants due to their role as a signaling molecule to act as a secondary messenger that regulates plant growth and development (Mustafavi et al., 2018; Jahan et al., 2021). On top of that, the stimulation of Put in plants is said to improve the stress tolerance of plants, protecting the plants against stress-induced damage (Collado-González et al., 2021). Jahan et al. (2021) investigated the combined effects of GSH and Put on three contrasting canola (*Brassica napus* L.) cultivars Shiralee, Dunkled, and Rainbow under hexavalent chromium Cr^6+^ stress. The combination of GSH and Put showed a significant decrease in bioaccumulation of Cr^6+^ in the shoots while an increase in minerals like Mg, P, Mn and endogenous GSH in stems, leaves and roots of *cv*. Shiralee, which leads to a greater yield when compared to the single application of GSH or Put. From the results, it can be concluded that *cv*. Shiralee was Cr^6+^-tolerant, followed by Dunkled, while Rainbow was Cr^6+^-sensitive (Jahan et al., 2021).

#### Botanical Extracts – *Moringa* Leaf Extract

*Moringa oleifera* (Lam.) leaf extract (MLE) is one of the most popular botanical extracts that showed great potential in increasing crop resistance to heavy metals and salinity (Abd El-Mageed et al., 2017; Rehman et al., 2021). Leaf anatomy on MLE revealed high antioxidant contents such as GSH, AsA, salicylic acid, DPPH radical-scavenging activities, an essential antioxidant in preventing lipid peroxidation; osmoprotectants such as soluble sugars and free proline; macronutrients like N, P, K, Ca, Mg; micronutrients like Fe, Cu, Zn, Mn; and phytohormones such as auxins, cytokinins and gibberellins (Abd El-Mageed et al., 2017). Abd El-Mageed et al. (2017) showed successful mitigation of drought and salinity-induced stress damage due to the role of MLE in regulating antioxidants, RWC and osmoprotectants.

Since MLE has proven to alleviate stresses successfully, the effects of the combined application of MLE and GSH on wheat (*Triticum aestivum* L.) in mitigating salinity stress were investigated (Rehman et al., 2021). The combination treatment had different sequences where seeds could be first soaked in 3% MLE for 12 h and sprayed with 1 mM of GSH 50 days after sowing (MLE-GSH), or seeds could be first soaked in 1 mM of GSH for 12 h and sprayed with 3% of MLE 50 days after sowing (GSH-MLE). Interestingly, the later treatment combination GSH-MLE resulted in the highest increase in growth traits such as shoot length, dry weight, leaf area, number of leaves, tillers per plant, and grain yield, due to their combined roles in increasing the total chlorophyll and carotenoid contents and increasing antioxidants such as GSH and AsA. GSH-MLE successfully alleviated the ionic and osmotic stress induced by salinity stress due to their combined role in regulating ionic homeostasis by increasing the accumulation of K^+^ and Ca^2+^ over Na^+^; increasing the RWC and MSI by reducing EL, and most accumulated osmoprotectants such as total soluble sugars (TSS) and free proline. The combined application of MLE and GSH in both sequences showed a significant improvement from the single application treatment groups (Rehman et al., 2021).

#### Inorganic Compound – Selenium

There are five primary plant growth-promoting elements, namely aluminum (Al), cobalt (Co), sodium (Na), selenium (Se), and silicon (Si), found in soils usually as inorganic salts or insoluble forms (du Jardin, 2015). Among the elements, the role of Se in alleviating stress-induced damage through activation of the antioxidant defense system in plants has been explored and studied (Chu et al., 2010; Jiang et al., 2017). These studies demonstrated the significance of Se as an antioxidant and a cofactor of the enzyme GPX, catalyzing the reduction of H_2_O_2_, which is overproduced when the plants are under stress (Elkhatib et al., 2021). Besides that, Se can control the permeability and changes in activities in a plant cellular membrane (Dawood et al., 2020). For instance, the application of Se maintained the ultrastructure of both chloroplast in mitochondria, further improving photosynthesis performance and salinity stress tolerance in sorrel seedlings (Kong et al., 2005). Dawood et al. (2020) demonstrated that the combined application of 300 mg/l of GSH and 10 mg/l of Se resulted in the highest increase in growth and yield parameters of all three wheat (*Triticum aestivum* L.) cultivars Egypt-2, Shandaweel-1, Gemmiza-11, under salinity stress of 3.95 dS/m, when compared to the single application of GSH or Se. The roles of both GSH and Se as antioxidants also gave the most tremendous increase in photosynthetic pigments (Chl a, Chl b, carotenoid) and improvement in the leaves and grains of wheat biochemical contents such as carbohydrate, flavonoid, phenolic and indole acetic acid (IAA), main auxin of plants (Dawood et al., 2020).

#### Humic Substances – Humic Acid

Humic substances such as humic and fulvic acid are plant and animal decomposed matter, formed by biochemical transformation and microbial metabolism, found readily in soils, which represents the central pool of organic carbon on the surface of the earth (Mackowiak et al., 2001; Canellas et al., 2015). Humic acids (HA) are humus materials that are aqueous-alkaline soluble and have been proposed as a sustainable alternative to alleviate salinity stress and improve plant crop productivity (Elkhatib et al., 2021). Previous studies have proved the significance of HA application in stimulating plant growth through the increase in root length, development of secondary shoots and higher uptake of nutrients. HA can also permanently tie up Na^+^ and Cl^−^ ions, increase plant membrane permeability and promote uptake of macronutrients like N, P, K, Ca, and Mg, which alleviates the ionic and osmotic stress induced by salinity stress (Mackowiak et al., 2001; Canellas et al., 2015; Elkhatib et al., 2021). Recently, Elkhatib et al. (2021) evaluated the effects of exogenous GSH, Se and HA individually and combined them as paired treatments (GSH + Se, Se + HA, and GSH + HA) on sweet pepper plants (*Capsicum annuum* L.) grown under salinity stress. All treatments individual or paired resulted in a significant improvement in vegetative growth and yield in plants under salinity stress, which was correlated to the increase in total chlorophyll, decrease in Na^+^ uptake, increase in nutrient uptake such as N, P and *Ca*. Among the individual treatments, Se recorded the best parameters followed by GSH and HA. As for the paired treatments, GSH + Se recorded the best parameters, followed by Se + HA and GSH + HA (Elkhatib et al., 2021).

#### Microorganism – Cyanobacteria

Cyanobacteria (CB), also known as blue-green algae, are photosynthetic prokaryotes that are ubiquitous components of the biocrust communities (Chamizo et al., 2018; Rai et al., 2019). CB is considered a natural biofertilizer that improves soil fertility due to its role in fixing carbon and nitrogen and excreting growth-promoting substances like hormones, vitamins, and amino acids (Chamizo et al., 2018; Rady et al., 2018). Upon their death and decomposition, CB is known to increase the water retention and biomass of soil. Furthermore, inoculation of CB reduces the content of oxidized matter in the soil and provides it to the submerged rhizospheres of plants, alleviating salinity stress and enhancing plant growth. Upon high salinity stress, CB restricts entry of Na^+^ ions, regulating the internal Na^+^ concentration, preventing oxidative damage to plants (Chamizo et al., 2018;Rady et al., 2018 ; Zaki et al., 2019).

Because of the beneficial roles of CB, especially in alleviating salinity stress of plants, the effects of combined application of CB and GSH on soybean (*Glycine max* L., cv. Giza 111) and common bean (*Phaseolus vulgaris* L., cv. Bronco) in mitigating salinity stress were investigated in two separate studies (Rady et al., 2018; Zaki et al., 2019). Zaki et al. (2019) showed that the combined application of CB and GSH resulted in the best growth characteristics and yield of salt-stressed soybean plants compared to the single application of CB or GSH. The results are correlated to the best improved photosynthetic efficiency, increased enzymatic activities of SOD, CAT and GPOX, increased relative water content, increased macronutrients like N, P, K^+^ and Ca^2+^ but reduced Na^+^ content and electrolyte leakage that successfully alleviates the salinity stress of soybean plants (Zaki et al., 2019).

In the other study, AsA was added to the combination of CB and GSH in two different sequences, CB + AsA + GSH and CB + GSH + AsA. The integrative CB + AsA + GSH application resulted in the best growth parameters: plant length, number of leaves per plant, leaf area per plant, fresh weight, and dry weight of plants. The positive effects of the combination CB + AsA + GSH treatment superior to a single application of GSH or AsA were attributed by the role of CB in improving soil characteristics, lowering the soil pH, and facilitating the available soil nutrients for plant uptake, as well as increasing the RWC and maintaining MSI. Adding CB to the antioxidants GSH and AsA has boosted the plant’s tolerance against salinity stress, coupled with the antioxidant effects in enhancing the enzymatic and photosynthetic activities, resulting in the best growth and yield of common bean plants (Rady et al., 2018).

## Conclusions and Future Perspectives

Undeniably, glutathione is the most pivotal metabolite in a plant’s antioxidant defense system and is vital for the improvement of the tolerance of plants against the high environmental stresses in recent days. The well-established understanding of biosynthesis and metabolism pathways of GSH demonstrates the importance of the GSH/GSSG redox ratio that functions as a stress signal to modulate responses during any stressed conditions. It is fascinating to observe the recent drastic progress in exploring of exogenous roles of GSH against the abiotic stress in the plant, pinpointing its compelling intrinsic value and potentials. In short, the exogenous GSH induces increased levels of endogenous GSH and improved GSH/GSSG ratio, which allows efficient scavenging of the overproduced ROS, alleviating the oxidative-induced stress and damage on plants. Moreover, exogenous GSH also induces an increased photosynthetic performance, which results in better vegetative growth and fruit yield in various plant crops in non-stressed and stressed conditions. Recent findings also suggested the significance of exogenous GSH in mitigating flood or submergence stress, relatively unexplored abiotic stress, and bacterial infection-induced stress, the first of the studied biotic stresses. Interestingly, in response to the biotic stress, the exogenous GSH induced similar responses to the abiotic stress, shown through the increase in photosynthetic performance, reduced ROS accumulation, and successfully ameliorated the biotic stress. In future, more studies on different types of biotic stresses or pathogenic diseases should be investigated to understand further and analyze the underlying mechanism of exogenous GSH in mitigating biotic stresses. In the sequenced or combined application, most bioactive compounds had strong antioxidant properties or served as co-enzymes that stimulated the antioxidant defense system, reducing the overproduced ROS and alleviating the abiotic stresses. The coupled effect of the different antioxidants resulted in the best growth and yield parameters compared to the individual application of GSH or the bioactive compound.

Although extensive studies on the effects of exogenous GSH have been performed, results on the stress responses were still differential across different studies, particularly the variable response of the antioxidant enzymatic activities, where some studies reported a decrease in enzymatic activities albeit an increase in GSH/GSSG ratio (Goodarzi et al., 2020). Thus, further study on the cellular mechanism regulating enzymes of GSH metabolism and degradation is required. Besides that, more studies should be performed on the absorption and transportation pathway of exogenous GSH through cell compartments such as mitochondria, cytoplasm, and chloroplast. Also, studies on the exact quantification and underlying mechanism on the limit of improvement subjected to the GSH/GSSG ratio should be carried out. On top of that, more studies on the individual and especially combined exogenous application of GSH with other bioactive compounds should be carried out extensively to validate the feasibility of the research findings as a sustainable alternative to commercialized fertilizer in a practical agricultural production of a larger scale.

## Author Contributions

YK: data curation, original draft preparation, writing revisions, editing, and visualization. SW and BG: writing – review and editing. NI, GZ, AD, SS, PP, and KT: resources and review. ST: supervision and project administration. All authors have read and agreed to the published version of the manuscript.

## Funding

This work was supported by Tropical Medicine and Biology Platform, School of Science and School of Engineering, Monash University Malaysia. The authors are grateful for the financial support from the Unit of Excellence in Research and Product Development of Coffee [UoE63004], the UNIt of Excellence on Clinical Outcomes Research and IntegratioN (UNICORN) [FF65-UoE005], University of Phayao, Thailand; Talent mobility program 2019, Office of the Higher Education Commission; and National Science Technology and Innovation Policy Office, Thailand.

## Conflict of Interest

The authors declare that the research was conducted in the absence of any commercial or financial relationships that could be construed as a potential conflict of interest.

## Publisher’s Note

All claims expressed in this article are solely those of the authors and do not necessarily represent those of their affiliated organizations, or those of the publisher, the editors and the reviewers. Any product that may be evaluated in this article, or claim that may be made by its manufacturer, is not guaranteed or endorsed by the publisher.

## Abbreviations

γ -ECS: γ -Glutamylcysteine synthetase
APX: Ascorbate peroxidase
AsA: Ascorbate or ascorbic acid
CA: Citric acid
CB: Cyanobacteria
CAT: Catalase
Chl a: Chlorophyll a
Chl b: Chlorophyll b
Chl a + b: Total chlorophyll
DHA: Dehydroascorbate
DHAR: Dehydroxyascorbate reductase
EL: Electrolyte leakage
F_v_/F_m_: Ratio of variable fluorescence over maximum fluorescence or maximum potential quantum efficiency of Photosystem II
Gly I: Glyoxalase I
Gly II: Glyoxalase II
GPX: Glutathione peroxidase
GR: Glutathione reductase
GSH: Glutathione or γ-glutamyl-cysteinyl-glycine or reduced glutathione
GSHS: Glutathione synthase
GSSG: Glutathione disulfide or oxidized glutathione
GST: Glutathione S-transferase
HA: Humic acid
HM: Heavy Metals
MDA: Malondialdehyde
MDHA: Monodehydroascorbate
MG: Methylglyoxal
MLE: *Moringa* Leaf Extract
MSI: Membrane stability index
MT: Melatonin
NADPH: Nicotinamide adenine dinucleotide phosphate
PA: Polyamine
PC: Phytochelatin
PCS: Phytochelatin synthase
POD: Peroxidase
Pro: Proline
Put: Putrescine
ROS: Reactive oxygen species
RWC: Relative water content
Se: Selenium
SLG: S-D-Lactoylglutathione
SOD: Superoxide dismutase
Spd: Spermidine
Spm: Spermine
Sub: Submergence
TCA: Tricarboxylic acid
