# Supplementary material for: Mitigation of Environmental Stress-Impacts in Plants: Role of Sole and Combinatory Exogenous Application of Glutathione
Source: Front Plant Sci. 2021 Dec 22;12:791205. doi: 10.3389/fpls.2021.791205 (PMC8728365; doi:10.3389/fpls.2021.791205)
Supplement: Supplementary file 1 [file Data_Sheet_1.docx]

# Supplementary Tables

**Supplementary Table 1**. Summary of results and effects of individual application of exogenous GSH on different plant species mitigating various stresses. Increased = ↑, decreased = ↓.

| Stress type | Stress level | Plant species | GSH application | Results and Effects | References |
| --- | --- | --- | --- | --- | --- |
| Heavy Metal Toxicity | 50 µM of Cadmium, Cd | Wheat (*Triticum aestivum* L. cv. Bainong 207) | 20 µM by foliar spray | - ↑ photosynthetic pigments: chlorophyll a (Chl a), chlorophyll b (Chl b) - ↑ Cd content in roots, ↓ Cd content in shoots, ↓ reduced translocation factor - ↓ MDA contents significantly - ↑ GST and GR activity - ↑ expression levels for GSH, GST, GR in roots and APX in shoots - ↓ expression levels of Cd transporter *TaNramp1, TaNramp5, TaHMA2, TaLCT1* for roots and shoots | (Li et al., 2021) |
|  | 15 µM of Arsenic, As (NaAsO) | Rice (*O. sativa* L. cv. Dasan) | 50 and 100 mg/kg by foliar spray | - 50 mg/kg of GSH resulted in the best parameters followed by 100 mg/kg of GSH - ↑ in plant height, shoot fresh weight and dry weight - ↓ As content in roots and shoots - ↓ activities of SOD, CAT in shoots, APX in leaves, ↑ MDHAR, DHAR and GR - ↑ AsA level, ↓ DHA level, ↑ AsA/DHA ratio - ↑ GSH level, ↓ GSSG level, ↑ GSH redox ratio - Effect of GSH was more evident in shoots than roots | (Jung et al., 2019) |
|  | 25 µM of Arsenic, As (NaAsO ), 500 µM L-buthionine sulfoximine (BSO) or 5 µM lycorine | Brinjal (*Solanum melongena* L. *cv.* Black Beauty) | 1 mM given to seedlings | - Mitigate As stress and adverse effects of lycorine and BSO (inhibitors of AsA and GSH) - Significantly ↓ As accumulation in leaf and roots cancelled the effect of cell death - ↑ level of endogenous NO and NOS-like activities - Alleviated protein damage, slightly ↑ O_2_^.-^ and H_2_O_2_ contents - ↑ GST, APX, MDHAR, DHAR and GR activities - Maintained the AsA/DHA and GSH/GSSG ratios | (Alamri et al., 2021) |
|  | 15 µM of Cadmium, Cd (CdCl_2_) | Maize (*Zea mays* L.) | 30 µM by foliar spray | - ↑ malic acid and maleic acid in leaves; decreased aconitic acid in roots, alleviate stress in TCA cycle - ↑ TCA cycle metabolites like heptanose, sophorose and sucrose in maize levels - ↑ osmotic metabolites like allo-inositol and decreased sorbitol content in leaves - ↑ flavonoids like caffeic acid and flavanone contents - ↑ consumption of ascorbic acid, alleviating oxidative stress - ↑ fatty-acid related metabolites such as linoleic acid, methyl linoleic acid, palmitic acid and stearic acid | (Wang et al., 2021) |
|  | 50 µM of Cadmium, Cd | Italian ryegrass (*Lolium multiflorum* cv. IdyII and Harukaze) | 200 µM GSH | - ↑ biomass of roots and shoots - ↑ stress tolerance index - ↑ Cd accumulation in roots but a decrease in shoots - ↓ Cd amounts in soluble fractions of leaf cells - ↓ Cd concentration extracted by ethanol - ↑ Cd concentration extracted by acetic acid | (Fang et al., 2020) |
|  | 1.0 mM of Pb(NO_3_)_2_ Lead, Pb | Wheat (*Triticum aestivum* L. cv. Pradip) | 1.0 mM by foliar spray | - ↓ amount of MDA, H_2_O_2_ and O_2_^-^ in the tissue and on the leaf surface - ↓ spots indicating a reduction of oxidative stress - ↑ endogenous AsA and GSH levels, reduced GSSG level, increased ratio of GSH/GSSG - ↑ activities of MDHAR, DHAR, GR, SOD, CAT, GPX - ↑ Gly I, Gly II activities, ↓ MG contents and proline contents - ↑ Chl a and Chl b contents - ↑ plant height, fresh weight, and dry weight. | (Hasanuzzaman et al., 2018) |
|  | 1000 mg/L Lead, Pb | *Iris lactea* var. *chinensis* | Long term: 100 - 1000 mg/L  Short term: 500 mg/L | - ↑ fresh weight in shoots and roots (1000 mg/L showed reduced increment) - ↑ GSH and PCs biosynthesis gene (*IIγ-ECS, IIGS, IIPCS*) expression level in shoots upon 24-hour exposure - ↑ IIγ-ECS and IIPCS expression level in roots upon 24-hour exposure - As opposed to other studies, ↑ Pb content in both shoots and roots - ↑ translocation rate of Pb (1000 mg/L showed reduced increment) - ↑ Pb tolerance in plants | (Yuan et al., 2018) |
| Salinity | 6.74 dS m^–1^ | Pepper (*Capsicum frutescence* (L.) | 0.4 and 0.8 mM by foliar spray every 14 days | - Significantly ↑ water use efficiency (WUE) - Significantly ↑ growth and yield parameters (number of branches, leaves, and fruits per plant, fresh and dry weight of shoots, roots, and fruits per plant) - ↑ contents of SPAD chlorophyll index, proline, phenols, capsaicin, AsA and GSH contents - ↑ leaf macro- (N, P, K^+^, Ca^2+^ and Mg^2+^), micro-nutrients and K^+^/Na^+^ ratio - ↑ values of osmoprotectant and antioxidant parameters | (Al-Elwany et al., 2020) |
|  | 100 mM NaCl and 1 mM BSO (glutathione inhibitor) | Tomato (*Solanum lycopersicum* L. cv. Zhongshu No. 4) | 5 mM by foliar spray | - Mitigated negative effects of NaCl and NaCl + BSO on relative growth rate - ↓ Na^+^ and Cl^-^, ↑ K^+^, Ca^2+^ and Mg^2+^ content in leaves and roots except for leaves at day 5 - Significantly ↓ the Na^+^/K^+^ and Na^+^/Ca^2+^ ratios in roots and leaves - ↓ the S_Na, K_ ratio on 5 d, the S_Na, Ca_ ratios at 10 and 15 d, and the S_Na, Mg_ ratio on the 10 d - ↓ polyamines (PA) like putrescine (Put), spermidine (Spd), and spermine (Spm) contents; ↑ (Spd + Spm)/Put ratios - ↑ PA degradation enzymes (PAO, ODC) activities - ↓ PA synthesis enzymes (DAO, SAMDC, ADC) activities - ↑ transcription levels of PAO, ODC but down-regulated DAO, SAMDC, ADC | (Zhou et al., 2018) |
| Chilling/ cold stress | 4 ± 0.5 °C | Bell pepper ([Capsicum annuum](https://www.sciencedirect.com/topics/food-science/green-bell-pepper) L.) | 0.05% (w/v) GSH by spraying | - Only mild CI symptoms were observed, CI incidence and index increased more slowly than the control group - No visible plasmolysis or mitochondrial swelling, cristae were intact, and chloroplasts had relatively normal spindle morphology, cellular integrity and ultrastructure were maintained - ↓ ROS levels, O_2_^-^, H_2_O_2_ and MDA content - ↑ AsA and GSH levels - ↑ APX, GR and MDHAR activities - ↑ AsA-GSH cycle genes CaAPX1, CaMDHAR1, and CaDHAR1 | (Yao et al., 2021) |
|  | 15 °C for 7 days | Rice (*Oryza sativa* L.) | 0.5 mM and 1.0 mM GSH by spraying | - ↑ endogenous GSH levels - ↑ in length and number of lateral roots and shoot height - ↑ transcription factors DREB and ERF; JA biosynthesis and signaling genes AOS, LOX and JAZ; auxin signaling gene SAUR - ↑ RNA-seq-based transcriptome, OsGS expression - ↓ ROS such as O^2 −^ and H_2_O_2_ | (Park et al., 2021) |
| Drought | 15% polyethylene glycol 6000 for 4 days | Rice (*Oryza sativa* L. cv. BRRI dhan29) | 0.2 mM GSH for 72 h | - ↑ root length, shoot and root dry and fresh weights of stressed seedlings - ↑ chlorophyll pigments (Chl a, Chl b, and total chlorophyll) - ↑ relative water content (RWC), total soluble sugars (TSS) but ↓ proline of stressed seedlings (different from other studies) - ↓ O_2_^.-^, H_2_O_2_ and MDA content in shoot and root tissues - ↑ CAT, APX and POX activities in shoots and roots - ↑ AsA and carotenoid content - ↑ uptake of Na^+^, K^+^, Ca^2+^ and Mg^2+^ ions | (Sohag et al., 2020) |
| Flood/ Submergence | Fully submerged in a pond for 14 days | Rice cultivars BRRI dhan29 and dhan52 | 1 and 2 mM by foliar spray on every second day for 2 weeks | - ↑ levels of chlorophylls (Chl a, Chl b, total chlorophyll), carotenoids, soluble proteins, and proline. - ↓ levels of H_2_O_2_ and malondialdehyde (MDA), mitigated Sub-induced oxidative damage - ↑ activities of antioxidant enzymes like SOD, APX, POD, CAT, GPX and GST - dhan52 was more tolerant to Sub-stress than dhan29 | (Siddiqui et al., 2021) |
| Bacteria | 10^-7^ CFU *X. campestris* | Chilli (*Capsicum annum* L. cv. Serrano and Desi) | 1.0 mM by seed priming | - Priming GSH before pathogen inoculation showed better parameters than priming after pathogen inoculation - Serrano is moderately susceptible, Desi is susceptible to bacterial spot disease - ↑ growth rate, plant dry weight with no and least symptoms of leaf spot disease - ↑ chlorophyll pigments such as Chl a and Chl b - ↑ most photosynthetic fluorescence parameters such as the efficiency of photosystem II (ΦPSII), non-photochemical quenching (NPQ), linear electron flow (LEF), and photochemical quenching (qP) - ↑ photon energy absorbed in PSII used for photosynthesis (P%) and thermal energy dissipated by PSII (D%) - Significantly ↓ concentration of H_2_O_2_ and MDA, reducing ROS. - ↑ endogenous GSH and GSH/GSSG ratio - ↑ activities of SOD, POD and CAT | (Ramzan et al., 2021) |

**Supplementary Table 2**. Summary of results and effects of combined application of exogenous GSH with other bioactive compounds on different plant species mitigating different stresses. Increased = ↑, decreased = ↓, best/highest = ⤒, lowest = ⤓

| Combined application | Stress | Method & dosage | Plant type | Results and Effects of Combined Application | References |
| --- | --- | --- | --- | --- | --- |
| GSH + Ascorbic acid (AsA) + Proline | Drought, irrigation level (7, 14, 28 days) | Seed soaking, AsA: 0.75mM for 90 mins,  GSH: 0.75mM for 80 mins,  Pro: 0.75mM for 70 mins | Chickpea (*Cicer aritinium* L.) | Combination of **GSH + AsA + Proline** showed the best parameters followed by individual application of GSH, proline, AsA and control:   - ⤒ growth parameters (shoot & root lengths, fresh and dry weight of shoots and roots) & yield attributes (no of pods/plant, weight of pods/plant, no of seeds/plant and weight of 100 seeds) - ⤒ increment in Chl a, Chl b, total chlorophyll, carotenoids - ⤒ endogenous proline, AsA, GSH and protein content - Significantly ⤒ the antioxidant enzyme activities (CAT, SOD, POX, APX and GR) in roots and leaves | (El-Beltagi et al., 2020) |
|  | Cadmium, Cd (2 mM of CdCl_2_) | Seed soaking, AsA: 0.5mM for 90 mins,  Pro: 0.5mM for 80 mins,  GSH: 0.5 mM for 70 mins | Cucumber (*Cucumis sativus* (L.)) | Sequenced treatment of **AsA-proline-GSH** showed the best parameters followed by individual application of GSH, AsA, proline and control:   - ⤒ transplant growth characteristics - ⤒ contents of proline, AsA and GSH - ⤒ enzymatic activities of SOD, CAT, GR and APX - ⤓ Cd^2+^ content in roots and leaves - ⤒ stomatal conductance, chlorophyll content and photosynthetic efficiency | (Semida et al., 2018) |
|  | Salinity (100 mM NaCl) | Seed soaking. 0.5 mM AsA for 90 min, 0.5 mM Pro for 80 min, 0.5 mM GSH for 70 min | Cucumber(*Cucumis sativus* (L.)) | Sequenced treatment of **AsA-proline-GSH** showed the best parameters followed by individual application of GSH, AsA, proline and control:   - ⤒ growth characteristics (shoot length, leaf area, stem diameter, shoot fresh and dry weight) - ⤒ stomatal conductance, chlorophyll, the maximum quantum yield of photosystem II (PSII) and photosynthetic efficiency - ⤒ relative water content (RWC), membrane stability index (MSI), free proline, AsA and GSH - ⤒ enzymatic activities of SOD, CAT, GR, APX - ⤓ Cd^2+^ contents in shoots and roots in stressed and non-stressed - ⤒ K^+^ and Ca^2+^ contents but lowest Na^+^ content in shoots and roots of the stressed condition - ⤒ K^+^/Na^+^ ratio and Ca^2+^/Na^+^ ratio in shoots and roots in the stressed condition | (Seleiman et al., 2020) |
|  | Salinity | Foliar spray. AsA-Pro-GSH and GSH-Pro-AsA each 0.5 mM repeated at 30 and 40 DAS | Faba bean (*Vicia faba* L.) | Sequenced treatment of **AsA-proline-GSH** showed the best parameters followed by individual treatment of GSH, sequenced treatment GSH-Pro-AsA, individual treatment of proline and control:   - ⤒ growth traits like shoot length, no. of leaves, brunches, shoot fresh weight, dry weight and leaves area - ⤒ relative chlorophyll content, photosynthetic efficiency (Fv/Fm, Fv/F0, PI), and stomatal conductance - ⤒ improvement in leaf RWC, MSI, water use efficiency (WUE) - ⤒ level in GR, CAT, SOD, and APX activities - ⤒ content of AsA and GSH. - ⤒ yield such as green pods weight per plant, green pods yield per hectare, and seed yield per hectare in the two seasons | (Semida et al., 2021) |
| GSH + Citric acid (CA) | Lead, Pb (300 & 600 mg/kg) | Foliar spray, CA (5mM), GSH (25mM) | Castor bean (*Ricinus communis* L.) | - ↑ length and dry weight of shoots and roots, leaf area, number of leaves per plant under non-stressed and stressed conditions. - ↑ Chl a, Chl b, total chlorophyll, carotenoids under non-stressed and stressed conditions - ↑ gas exchange parameters with the maximum escalation in the water use efficiency, rate of transpiration, stomatal conductance, and photosynthetic rate under non-stressed and stressed conditions - ↓ malondialdehyde (MDA), electrolyte leakage (EL) and hydrogen peroxide (H_2_O_2_) in the leaves and roots - ↑antioxidant enzyme activities (SOD, POD, CAT, APX) in roots and leaves | (Zeng et al., 2021) |
| GSH + Melatonin (MT) | Zinc, Zn (500 μm) | Root irrigation: GSH (100 μm); Foliar spray:  MT (100 μm) | Safflower (*Carthamus tinctorius* L.) | Combination treatment of **GSH+MT** resulted in the best parameters followed by individual treatment of GSH, MT and control:   - ⤒ root and shoot dry weight and total chlorophyll - ⤓ Zn concentration in roots and shoots when compared to singly applied GSH, MT and control under stressed conditions - ⤓ accumulation of MDA, H_2_O_2_ and LOX activity in the leaves and roots - ⤒ AsA/DHA ratio and GSH/GSSG ratio but behind the single application of GSH - ⤒ total phytochelatin (PC) content in shoots - ⤒ CAT and DHAR; ↓SOD, APX, MDHAR, GR and both glyoxalase activities (Gly I and Gly II) as opposed to other studies | (Goodarzi et al., 2020) |
| GSH + Putrescine (PUT) | Chromium, Cr^6+^ (50 μg/g K_2_Cr_2_O_7_) | Foliar spray: GSH 0.1 mM, PUT 1 mM | Canola (*Brassica napus* L. cvs. Shiralee, Rainbow, and Dunkled) | - Effects of combination treatment improved agronomic performance but were differential and specific to cultivars, cv. Shiralee is Cr^6+^ tolerant, cv. Dunkled is moderately sensitive, cv. Rainbow is Cr^6+^ sensitive - ↑ number of pods in *cv.* Dunkled significantly as compared to a single application of GSH and PUT - ↑ endogenous GSH in roots and leaf in *cvs.* Rainbow and Dunkled - ↓ leaf NO content in *cv.* Rainbow - ↑ root Cr^6+^ bioaccumulation in *cv.* Shiralee and Rainbow - ↑ minerals (Mg, P, S, Fe, Mn, Zn) in roots and shoots differentially - ↑ number of pods per plant, pod length, no. of seeds per pod and weight of seeds differentially | (Jahan et al., 2021) |
| GSH + *Moringa oleifera* leave extract (MLE) | Salinity 9.10 dS/m | Seed soaking for 12 hours and foliar spray 50 DAS: MKE 3% and GSH (1mM) | Wheat (*Triticum aestivum* L.) | Combination of **GSH-soaked followed by MLE-sprayed** resulted in the best parameters followed by MLE-MLE, MLE-GSH, GSH-GSH and control:   - ⤒ plant growth (shoot length, shoot dry weight, leaf area, number of leaves and tillers per plant) under stressed and non-stressed conditions - ⤒ membrane stability index (MSI) and leaf relative water contents (RWC) by reducing electrolyte leakage (EL) - ⤒ total chlorophyll and carotenoid contents - ⤒ accumulation of osmoprotectants (free proline, total soluble sugars (TSS)) and non-enzymatic antioxidants (ascorbate (AsA), glutathione (GSH)) - ⤒ ionic homeostasis: ⤒ accumulation of K^+^ and Ca^2+^ over Na^+^, ⤒ ratio of K^+^/Na^+^, Ca^2+^/Na^+^, K^+^+Ca^2+^/Na^+^) in wheat leaves by regulating their uptake - ⤒ hormones contents: indole 3 acetic acid (IAA), gibberellic acid (GA3), Zeatin) and ⤓ abscisic acid (ABA) contents - ⤒ grain yield under stressed and non-stressed conditions | (Rehman et al., 2021) |
| GSH+ Selenium (Se) | Salinity, 3.95 dS/m | GSH (200, 300 mg/l) Se (10 mg/l) | Wheat (*Triticum aestivum* L.) | Combination treatment of **GSH + Se** resulted in the best parameters followed by individual treatment of Se, GSH and control:   - ⤒ shoot height, leaves number/teller, and fresh and dry weight of shoot of three wheat cultivars - ⤒ total photosynthetic pigments. - ⤒ shoot height, spike length, number of spikelets/spikes, biological yield/teller, grain yield/teller, straw yield/teller, grains index, biological yield, grain yield (ton/fed). - ⤒ Indole Acetic Acid (IAA), carbohydrate content, flavonoid, and phenolic content | (Dawood et al., 2020) |
| GSH + Selenium,  GSH + Humic acid | Salinity, 500, 1000, 1500 ppm | Foliar spray: GSH (25 mg/L), Se (5 mg/L), HA (200 mg/kg soil) | Sweet pepper (*Capsicum annuum* L.) | Sequenced treatment of **GSH + Se** resulted in the best parameters followed by GSH+HA, Se+HA and control:   - ⤒ vegetative growth parameters (leaves fresh & dry weight, leaf area per plant under non-stressed and stressed conditions. - ⤒ total chlorophyll content in leaves - ⤒ N, P and Ca contents in leaves - ⤒ K^+^ uptake, ↓ Na^+^ uptake, ⤒ K^+^/Na^+^ ratio - ⤒ yield per plant | (Elkhatib et al., 2021) |
| GSH + Cyanobacteria (CB) | Salinity, 150 mM | Seed inoculation with CB, Foliar spray: GSH (1 mM) | Soybean (*Glycine max* L., cv. Giza 111) | Combination treatment of **GSH + CB** resulted in the best parameters followed by individual treatment of CB, GSH and control:   - ⤒ growth characteristics and yield components - ⤒ photosynthetic efficiency, pigments contents and chlorophyll fluorescence - ⤒ membrane stability index (MSI), relative water content (RWC) - ⤒ soluble sugars, free proline, AsA, GSH, α-tocopherol, and protein - ⤒ activities of SOD, CAT, and GPX - ⤒ contents of macronutrients (N, P, K^+^, and Ca^2+^) - ⤓ Na^+^ content and electrolyte leakage (EL) | (Zaki et al., 2019) |
| CB + GSH/ CB + GSH + AsA | Salinity, 7.35-7.42 dS/m | Seed inoculation with CB, Foliar spray: AsA (1.0 mM), GSH (0.75 mM) | Common bean (*Phaseolus vulgaris* L., cv. Bronco) | Sequenced treatment of **CB-AsA-GSH** resulted in the best parameters followed by treatment CB-GSH-AsA, CB-GSH, CB-AsA, individual treatment of GSH, AsA and control:   - ⤒ plant length, number and area of leaves, plant fresh and dry weights - ⤒ yield parameters such as green pods weight per plant, dry seed weight per plant and 100-seed weight - ⤒ [photosynthetic leaf pigments](https://www.sciencedirect.com/topics/agricultural-and-biological-sciences/photosynthetic-pigment) and photochemical efficiency (Fv/Fm) - ⤒ relative water content and membrane stability index - ⤒ contents of soluble sugar, [proline](https://www.sciencedirect.com/topics/agricultural-and-biological-sciences/proline), AsA, GSH - ⤒ N, P and K^+^ ion contents - ⤒ activities of SOD, CAT and GPX - ⤓ Na^+^ ion content and electron leakage (EL) | (Rady et al., 2018) |
